# Therapeutic Uses and Pharmacological Properties of Shallot (*Allium ascalonicum*): A Systematic Review

**DOI:** 10.3389/fnut.2022.903686

**Published:** 2022-07-28

**Authors:** Cadmiel Moldovan, Oleg Frumuzachi, Mihai Babotă, Lillian Barros, Andrei Mocan, Simone Carradori, Gianina Crişan

**Affiliations:** ^1^Pharmaceutical Botany Department, Faculty of Pharmacy, “Iuliu Haţieganu” University of Medicine and Pharmacy, Cluj-Napoca, Romania; ^2^Centro de Investigação de Montanha (CIMO), Instituto Politécnico de Bragança, Bragança, Portugal; ^3^Laboratory of Chromatography, Institute of Advanced Horticulture Research of Transylvania, University of Agricultural Sciences and Veterinary Medicine, Cluj-Napoca, Romania; ^4^Department of Pharmacy, “G. d'Annunzio” University of Chieti-Pescara, Chieti, Italy

**Keywords:** shallot, *Allium ascalonicum*, *Allium cepa* var. *aggregatum*, pharmacological activity, therapeutic use

## Abstract

**Background:**

Shallot (*Allium ascalonicum* L.) is a traditional plant species used throughout the world both for culinary purposes and as a folk remedy. To date (i.e., April 2022), there is no report on the main pharmacological activities exerted by shallot preparations and/or extracts.

**Scope and Approach:**

The aim of this study was to comprehensively review the pharmacological activities exerted by shallot, with rigorous inclusion and exclusion criteria based on the scientific rigor of studies. Prisma guidelines were followed to perform the literature search.

**Key Findings and Conclusions:**

The literature search yielded 2,410 articles of which 116 passed the required rigorous criteria for inclusion in this review. The extracts exert a potent antioxidant activity both *in vitro* and *in vivo*, as well as a strong inhibitory capacity on various pathogens with relevant implications for public health. Moreover, shallot can be used as adjuvant therapy in cardiovascular diseases, diabetes, cancer prevention, and other non-communicable diseases associated with inflammatory and oxidative pathways. Future studies investigating the chemical composition of this species, as well as the molecular mechanisms involved in the empirically observed pharmacological actions are required.

## Introduction

Medicinal plant extracts and their constituents possess various biological activities including antimicrobial, anti-inflammatory, antioxidant, cytotoxic, antidiabetic, and other activities (1).

*Allium* vegetables, including garlic, onion and shallot, are widely used as herbal supplements (2). Known as bulb crops, they make up specialized culinary ingredients owing to their characteristic aroma arising from organic sulfur compounds released from the enzymatic breakdown (by allinase and lachrymatory factor synthase) of the flavor precursors [*S*-alk(en)yl cysteine sulfoxide compounds] found in their cells (3). Sulfur compounds released from their decomposition and other bioactive compounds (e.g., flavonols and other phenols) exert prophylactic and curative properties against many human ailments like microbial infections, carcinogenesis and degenerative diseases. The commercial commodity obtained from *Allium* vegetables is commonly consumed afresh as well as processed in almost all cultures of the world, making alliums have an “all-rounder” presence in routine human life. Amongst the vast range of *Allium* species found in nature, shallot is one of the most economically important, used mainly in culinary preparations. The countries with the highest volumes of onion and shallot consumption in 2018 were China (25 M tons), India (20 M tons) and Pakistan (2.1 M tons), together comprising 80% of total consumption. The countries with the highest volumes of onion and shallot production in 2018 were China (26 M tons), India (22 M tons) and Pakistan (2.1 M tons), with a combined 84% share of total production (4).

Shallot (*Allium ascalonicum* L. or *Allium cepa*, Aggregatum group, *A. cepa* var. *aggregatum*) is an annual herbaceous plant of the family Amaryllidaceae that grows in various parts of the world (5).

### History

According to some scholars, shallot was brought to Europe by people who migrated from the Middle East, including the Celtic populations (6). The ancient Greeks gave shallot its name when their traders discovered it in the ancient Palestinian port of Ashkalon (now Ashkelon in Israel) and named it after the city (7). The word “shallot” comes from the Old French “escalogne,” from the Latin “Ascalonia caepa” (onion of Ashkalon).

### Description

Shallot is a plant that can reach 20–30 cm in height. It has cylindrical leaves, with a composite bulb (not unique), tunicate, often composed of two or three smaller cloves united in a single tunicate bulb slightly larger, on the whole slightly more tapered than the onion. This bulb normally has a weight that varies from about 5 to 25 grams and which, depending on the variety, differs in the color of the outer sheaths (purple-green, red, red-brown, pinkish-red, purple, yellow, gray, and white), of their shape (spherical, rounded and elongated) and finally of the taste, influenced by the cultivation area (8).

### Traditional Uses

While shallots are closely associated with traditional French recipes, such as Beef Bourguignon, they are truly international vegetables. Shallots are an authentic ingredient of many Asian cuisines, from Thai soups and red and green curries to Indonesian and fried rice dishes such as Nasi goring. According to a study conducted by Srisawat et al. in Nearby Khao Luang Mountain Hill Region, Southern Thailand, the shallot is used as a traditional remedy for lipoma. The leaves of shallot are pounded and administrated as a poultice, or used externally (9). Also, it was reported that in Ibarapa Local Government Area of Oyo State, Nigeria shallot's local name is “Alubosa elewe” and is used with a high frequency to treat onchocerciasis, a neglected tropical disease listed by the World Health Organization (10).

### Aim of the Study

Hundreds of laboratory studies have been conducted on this species. However, a comprehensive review, with rigorous inclusion and exclusion criteria based on the scientific rigor of studies, has never been attempted before.

Given the multitude of research papers published concerning this species as well as the promising pharmacological application of the extracts enriched in biologically active compounds, this is a timely review on this topic.

Here, we present a comprehensive analysis of the literature studying *A. ascalonicum* L. species by focusing on its pharmacological activity.

## Materials and Methods

### Botanical Terminology

The plant name was cross-checked for accuracy in accordance with the Plant List (http://www.theplantlist.org/), the International Plant Names Index (http://www.ipni.org/index.html), and the World Checklist of Selected Plant Families (https://wcsp.science.kew.org/). Any botanical synonyms or citations with unaccepted author epithets were explicitly mentioned in the material data tables and manuscript text.

### Literature Search

We followed the PRISMA guidelines to perform our literature search (11). We systematically assessed the scientific literature collected from the Web of Science, PubMed, and Scopus scientific databases, considering all the articles published between 1973 and 2021 (April 20, 2022). The keywords “(‘Allium' AND ‘ascalonicum') OR (‘Shallot') OR (‘Allium' AND ‘aggregatum')” were used.

### Inclusion and Exclusion Criteria

Article titles and abstracts were manually screened to exclude studies not related to the topic. Only articles related to *A. ascalonicum* and/or *A. cepa* var. *aggregatum* were included in the analysis. Relevant articles were examined to determine if they fit the eligibility criteria of this review.

The specific inclusion criteria were as follows:

- Access to the full-text article in the English language. Articles published only in non-English languages were excluded.
- Validated source of material: plant materials were identified to the taxonomic level of genus and species with plant voucher specimens collected and deposited in a herbarium, university, or research institute.

The specific exclusion criteria were as follows:

- Articles which do not report the pharmacological action exerted by shallot
- Conference abstracts
- Articles with no full text available

### Data Analysis

Data obtained from the literature search was maintained and organized in Microsoft Excel, then analyzed and visualized manually using Microsoft PowerPoint.

## Results and Discussion

### Literature Assessment

**Figure 1:**
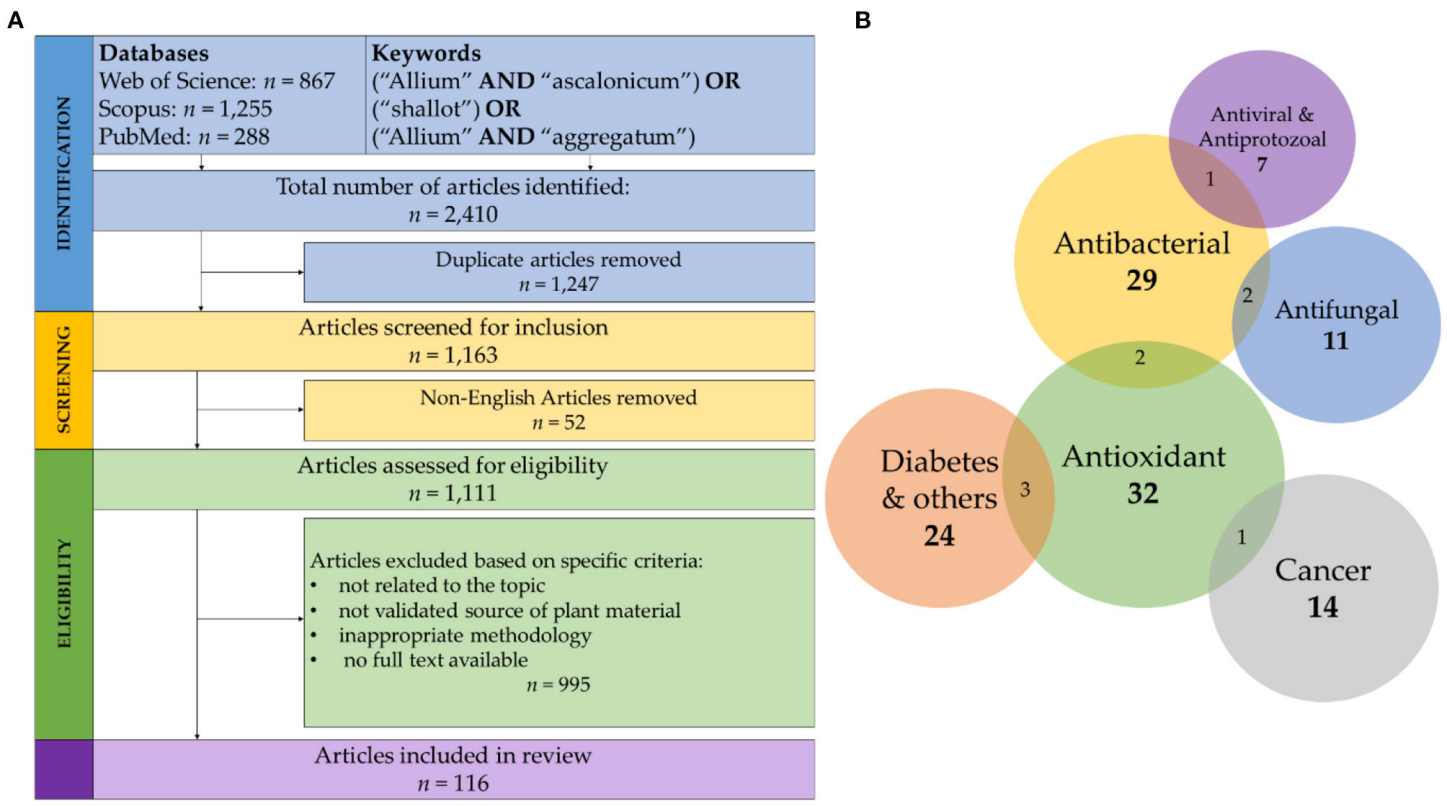
**(A)** Representation of inclusion and exclusion criteria, with corresponding results from the literature search used. **(B)** Venn Diagram of type and number of studies included in this review. The overlapping zones and the associated numbers correspond to number of articles which describe two or more pharmacological actions.

The process of identification and screening of articles for this review is represented in Figure 1A. The literature search yielded 2,410 articles from which 1,247 duplicates were excluded. Of these 1,111 publications, only 116, or ~10%, passed the rigorous criteria required for inclusion in this review.

In the following sections, we provide an overview of pharmacological actions exerted by extracts derived from shallot. Each section is accompanied by a table, where specific details are presented. Figure 1B provides an overview of the number of studies presented in this review.

## Antimicrobial Activity

The antimicrobial activity of *Allium* species has been long recognized, with allicin and other thiosulfinates, and their transformation products exhibiting a marked antimicrobial activity (12).

The compounds found in *Allium* extracts possess inhibitory activity on a broad range of microorganisms, such as bacteria, fungi, viruses, and parasites. Allium-derived antimicrobial compounds inhibit microorganisms by reacting with the sulfhydryl (SH) groups of target proteins. In this section, the documented antimicrobial effects of shallot will be discussed in detail. The section is divided into four main sub-sections (i.e., antibacterial, antiviral, antifungal, and antiparasitic/antiprotozoal activity).

### Antibacterial Activity

One of the first studies which assessed the antimicrobial capacity of shallot revealed that its juice does have no effect on inhibiting gram-negative bacteria on an agar diffusion test (13). However, over the past years, new data came across showing the capacity of shallot to inhibit a wide range of bacteria, both gram-negative and gram-positive, irrespective of the solvent used for the extraction of shallot compounds (ethanol, methanol, or water). Table 1 comprehensively summarizes, to our knowledge, the main findings related to bacterial inhibition exerted by different shallot extracts.

**Table 1:** Summary of main antibacterial, antifungal, antiprotozoal, and antiviral activity exerted by shallot extracts.

**Species**	**Bacterial strain**	**Inhibition capacity**	**Other species/conditions**	**Pre-treatment and extraction procedure**	**Zone**	**Species**	**References**
**Antibacterial activity**							
**Gram-positive**							
*Streptococcus agalactiae*	DMST1729	**E**: 14.67 mm (75 mg/ml)	*Tagetes erecta; Morus indica*	**D** (65°C, 24 h) >> **E** (EtOH 100%; SLR 1:5; 24 h) >> **Ev** (60 rpm, 289 mbar; 65°C)	Maha Sarakham, Thailand	Red shallot; *Allium ascalonicum;* bulb	(14)
*Streptococcus pyogenes*	ATCC19615	**E:** 8.67 (75 mg/ml)	*Tagetes erecta; Morus indica*	**D** (65°C, 24 h) >> **E** (EtOH 100%; SLR 1:5; 24 h) >> **Ev** (60 rpm, 289 mbar; 65°C)	Maha Sarakham, Thailand	Red shallot; *A. ascalonicum:* bulb	(15)
*Staphylococcus aureus*		**UAE**: MIC = 3.18 μg/ml	NA	**UAE** (Water; SLR 1:4; 10 min; 200 watts) >> **F** >> **C** (4,000 rpm;10 min) >> **FD**	Hamadan city, Iran	Shallot; *A. ascalonicum*; bulb	(16)
		**E**: MIC *=* 75 μg/ml	pH (4–11); 0–121°C; detergents; enzymes	**E** (Water; SLR 2:1; 5 h) >> **F** >> **D** (50°C) >> **Fract**: silica gel column (12–15/40)	NA	Shallot; *A. ascalonicum;* bulb	(17)
		**B**: 7.42; **E**: 6.33; **SD 5** **μM**: 3.65; **SD 10** **μM**: 2.57 [log CFU/ml]	Shallot? *(A. fistulosum)*	**E** (Water; SLR 1:1; 5 min; 25°C) >> **F** **SD** (3 h)	Local farms	Scallion?; *A. ascalonicum*; edible portion	(18)
		**UAE**: 1.1 mm **dd** = 0 mm	*A. sativum*	**UAE** (Water; SLR 1:10; 30°C; 15 min; 20 kHz) >> **C** (3,000 × g; 15 min; 4°C) >> **F**	Nha Trang, Vietnam	Shallot; *A. ascalonicum*; bulb	(19)
	ATCC 25923	**E**: MIC = 25,000; MMC = 200,000 **PC:** MIC = 0.625; MMC = 5	NA	**E** (EtOH 95%; SLR 1:1; one night, r.t.) >> **Ev** >> **FD** **PC:** Gentamycin	Thai market, Pathumthani, Thailand	Shallot; *A. ascalonicum* Linn.; bulb	(20)
	ATCC 25923	**SD**: 16.0 ± 0.1 mm **PC**: 26.6 ± 0.6 mm **dd** = 6.0 mm	*A. sativum; A. cepa; A. porrum; A. tuberosum; A. schoenoprasum*	**SD** (turbo hydrodistillation; Water; SLR 1:1.6) >> +petroleum ether >> **F** >> **Ev** (40°C) (y = 80–120 mg/kg); 15 μl **PC**: 30 μg amoxicillin/clavulanic acid	Avignon province, France	Shallot; *A. ascalonicum*; bulb	(21)
	ATCC 6538	**SD**: MIC = 150; MBC = 600	*##*	**D** (r.t., dark) >> **SD** (2 h); pH = 6; t = 8°C	West Azarbyjan, Iran	NA; *A. ascalonicum* L.; NA	(22)
	ATCC25923	**PC:** 15 mm; **J**: 13 mm; **Soxtec:** 24 mm; **dd** = 6 mm	#	**J** (20 g); **Soxtec** **PC**: Gentamycin;	Bangkok, Thailand	Shallot; *A. ascalonicum* L.; NA	(23)
	*MRSA*	**B**: 8.14; **E**: 7.34; **SD 5** **μM**: 3.35; **SD 10** **μM**: 1.82; (log CFU/ml)	Shallot?; *(A. fistulosum)*	**E** (Water; SLR 1:1; 5 min; 25°C) >> **F; SD** (3 h)	Local farms	Scallion?; *A. ascalonicum*; edible portion	(18)
	*ATCC 25923*	**E**: 512 μg/ml	Irradiation dosage (1–9 Gy)	**E** (EtOH 80%; SLR 1:30; 24 h, 200 rpm, r.t.) >> **IRR** (at 2 and 6 Gy)	Samosir farm, Indonesia	*Allium cepa* L. var. *ascalonicum* Backer	(24)
*Staphylococcus epidermidis*		**E**: MIC = 50,000; MMC = 100,000; **PC:** MIC = 1.250; MMC = 1.250	NA	**E** (EtOH 95%; SLR 1:1; one night, r.t.) >> **Ev** >> **FD** **PC:** Gentamycin	Pathumthani, Thailand	Shallot; *A. ascalonicum* Linn.; bulb	(20)
*Lactobacillus* sp.		**Frying**: 1.87 mm; **J:** 0.54 mm; **dd** = 0 mm	NA	**Frying** >> Water by-product **>>** **D** (r.t.); **J** (Water, SLR = 2:1) >> **E** (EtOH 98%, 3 days) >> **F** >> **FD**	Palu, Indonesia	Shallot; *A. ascalonicum;* Frying by-product	(25)
*Bacillus subtilis*	ATCC 6633	**E**: MIC = 25,000; MMC = 25,000; **PC:** MIC = 1.250; MMC = 1.250	NA	**E** (EtOH 95%; SLR 1:1; one night, r.t.) >> **Ev** >> **FD** **PC:** Gentamycin	Thai market, Pathumthani, Thailand	Shallot; *A. ascalonicum* Linn.; bulb	(20)
		**E**: MIC *=* 38 μg/ml	pH (4–11); 0–121°C; detergents; enzymes	**E** (Water; SLR 2:1; 5 h) >> **F** >> **D** (50°C) >> **Fract**: silica gel column (12–15/40)	NA	Shallot; *A. ascalonicum;* bulb	(17)
*Bacillus cereus*		**E**: MIC = 5,000–10,000; MBC = 5,000		**E** (Water; SLR 2:1, 5 h) >> **F**	Zagros mountains, Iran	Shallot/red onion; *A. ascalonicum;* bulb	(26)
	*ATCC25923*	**PC:** 32 mm; **J:** 9 mm; **Soxtec**: 13 mm; **dd** = 6 mm;	#	**J** (20 g); **Soxtec** **PC**: chloramphenicol	Local market, Bangkok, Thailand	Shallot; *A. ascalonicum* L.; NA	(23)
		**UAE**: 0.4 mm; **dd** = 0 mm	*A. sativum*	**UAE** (Water; SLR = 1:10; 30°C; 15 min; 20 kHz) >> **C** (3,000 × g; 15 min; 4°C) >> **F**	Nha Trang, Vietnam	Shallot; *A. ascalonicum*; bulb	(19)
*Listeria monocytogenes*		**B**: 7.53 ± 0.17; **E**: 6.92 ± 0.12; **SD 5** **μM**: 3.04 ± 0.11; **SD 10** **μM**: 1.91 ± 0.10; (log CFU/ml)	Shallot?; *(A. fistulosum)*	**E** (Water; SLR 1:1; 5 min; 25°C) >> **F; SD** (3 h)	Local farms	Scallion?; *A. ascalonicum*; edible portion	(18)
	*ATCC 19118*	**SD**: MIC = 600; MBC = 4,800	*##*	**D** (r.t., dark) >> **SD** (2 h); pH = 6; t = 8°C	West Azarbyjan, Iran	NA; *A. ascalonicum* L.; NA	(22)
*Mycobacterium fortuitum*	*ATCC 684*	**Soxhlet**: MIC = 100,000; MBC = 200,000; **PC**: MIC = 40; MBC = 40	Solvent (MeOH, *n*-hexane)	**D** (r.t.) >>**Soxhlet** (MeOH) >> **F** >> **Ev** **PC**: rifampicin	Ogbomoso area, Oyo, Nigeria	NA; *A. ascalonicum* L.; whole plant	(27)
*Mycobacterium smegmatis*	*ATCC 19420*	**Soxhlet**: MIC = 100,000; MBC = 200,000; **PC**: MIC = 40; MBC = 40	Solvent (MeOH, *n*-hexane)	**D** (r.t.) >>**Soxhlet** (MeOH) >> **F** >> **Ev** **PC**: rifampicin	Ogbomoso area, Oyo, Nigeria	NA; *A. ascalonicum* L.; whole plant	(27)
		**E:** MIC: 250; PC: <0.49;7.81	Solvent (chloroform, EtOH, MeOH, Water)	**E (**EtOH**)** **>** **F** **>** **Ev** **PC:** ciprofloxacin, rifampicin	Volta region. Ghana	Ghanaian shallot; *A. cepa* var *aggregatum*	(28)
*Mycobacterium phlei*	*ATCC 19420*	**Soxhlet**: MIC = 100,000; MBC = 200,000; **PC**: MIC = 40; MBC = 40	Solvent (MeOH, *n*-hexane)	**D** (r.t.) >>**Soxhlet** (MeOH) >> **F** >> **Ev** **PC**: rifampicin	Ogbomoso area, Oyo, Nigeria	NA; *A. ascalonicum* L.; whole plant	(27)
*Mycobacterium tuberculosis*		**E**: MIC = 500 μg/ml	NA	**E** (Ethyl acetate; SLR 1:1; 5 h) >> **F** >> **C** (5,000 rpm; 10 min) >> **Ev** (50°C)	Zagros mountains, Iran	NA; *A. ascalonicum;* underground root	(29)
*Micrococcus* sp.		**E**: 7.5 ± 0.23 mm	*A. cepa; A. sativum*	**E** (MeOH; SLR 1:2; 11 min; 4°C) >> F (cheesecloth) >> **E** (MeOH)	NA	Shallot; *A. ascalonicum*; tissues	(30)
				**Gram-Negative**			
*Escherichia coli*		**UAE**: MIC = 3.29 μg/ml	NA	**UAE** (Water; SLR 1:4; 10 min; 200 watts) >> **F** >> **C** (4,000 rpm;10 min) >> **FD**	Hamadan city, Iran	Shallot; *A. ascalonicum;* bulb	(16)
		**Frying**: 4.41 mm; **J:** 0.54 mm; **dd** = 0 mm	NA	**Frying** >> Water by-product **>>** **D** (r.t.); **J** (Water, SLR = 2:1) >> **E** (EtOH 98%, 3 days) >> **F** >> **FD**	Palu, Indonesia	Shallot; *A. ascalonicum;* Frying by-product	(25)
		**E**: MIC = 156.2 μg/ml	pH (4–11); 0–121°C; detergents; enzymes	**E** (Water; SLR 2:1; 5 h) >> **F** >> **D** (50°C) >> **Fract**: silica gel column (12–15/40)	NA	Shallot; *A. ascalonicum;* bulb	(17)
	ATCC 43894	**SD**: MIC = 1200; MBC = 4800	**##**	**D** (r.t., dark) >> **SD** (2 h); pH = 6; t = 8°C	West Azarbyjan, Iran	NA; *A. ascalonicum* L.; NA	(22)
		**E**: MIC = 20,000; MBC = 20,000	NA	**E** (Water; SLR 2:1, 5 h) >> **F**	Zagros mountains, Iran	Shallot/red onion; *A. ascalonicum;* bulb	(26)
	O157:H7	**B**: 8.11 ± 0.15; **E**: 7.08 ± 0.14; **SD 5** **μM**: 3.48 ± 0.09; **SD 10** **μM**: 2.19 ± 0.07; (log CFU/ml)	Shallot?; *(A. fistulosum)*	**E** (Water; SLR 1:1; 5 min; 25°C) >> **F; SD** (3 h)	Local farms	Scallion?; *A. ascalonicum*; edible portion	(18)
		**UAE**: 2.3 mm; **dd** = 0 mm	*A. sativum*	**UAE** (Water; SLR = 1:10; 30°C; 15 min; 20 kHz) >> **C** (3,000 × g; 15 min; 4°C) >> **F**	Nha Trang, Vietnam	Shallot; *A. ascalonicum*; bulb	(19)
*Proteus*		**UAE**: MIC = 7.33 μg/ml	NA	**UAE** (Water; SLR 1:4; 10 min; 200 watts) >> **F** >> **C** (4,000 rpm;10 min) >> **FD**	Hamadan city, Iran	Shallot; *A. ascalonicum;* bulb	(16)
*Proteus mirabilis*		**E**: MIC = 10,000; MBC = 20,000	NA	**E** (Water; SLR 2:1, 5 h) >> **F**	Zagros mountains, Iran	Shallot/red onion; *A. ascalonicum;* bulb	(26)
*Pseudomonas*		**UAE**: MIC = 7.57 μg/ml	NA	**UAE** (Water; SLR 1:4; 10 min; 200 watts) >> **F** >> **C** (4,000 rpm;10 min) >> **FD**	Hamadan city, Iran	Shallot; *A. ascalonicum;* bulb	(16)
		Bacteria count < 6 log10 cfu/g	*Trachyspermum ammi*	**D** (50°C) >> **E** (EtOH 95%; SLR 1:10, 24 h) >> **F** >> **FD** (30°C)	Gorgan, Iran	Shallot; *A. ascalonicum* L.; fruit	(31)
*Pseudomonas aeruginosa*		**E:** MIC = NE; MMC = NE; **PC:** MIC = 0.625; MMC = 5	NA	**E** (EtOH 95%; SLR 1:1; one night, r.t.) >> **Ev** >> **FD** **PC:** Gentamycin	Thai market, Pathumthani, Thailand	Shallot; *A. ascalonicum* Linn.; bulb	(20)
		**B**: 8.31 ± 0.16; **E**: 7.21 ± 0.18; **SD 5** **μM**: 4.62 ± 0.15; **SD 10** **μM**: 2.74 ± 0.06; (log CFU/ml)	Shallot?; *(A. fistulosum)*	**E** (Water; SLR 1:1; 5 min; 25°C) >> **F; SD** (3 h)	Local farms	Scallion?; *A. ascalonicum*; edible portion	(18)
*Klebsiella* sp.		**UAE**: MIC = 3.6 μg/ml	NA	**UAE** (Water; SLR 1:4; 10 min; 200 watts) >> **F** >> **C** (4,000 rpm;10 min) >> **FD**	Hamadan city, Iran	Shallot; *A. ascalonicum;* bulb	(16)
		**E**: 6.5 ± 0.28 mm	*A. cepa; A. sativum*	**E** (MeOH; SLR 1:2; 11 min; 4°C) >> **F** (cheesecloth) >> **E** (MeOH)	NA	Shallot; *A. ascalonicum*; tissues	(30)
*Klebsiella pneumonia*e		**B**: 8.27 ± 0.17; **E**: 6.72 ± 0.10; **SD 5** **μM**: 4.28 ± 0.18; **SD 10** **μM**: 2.53 ± 0.10; (log CFU/ml)	Shallot?; *(A. fistulosum)*	**E** (Water; SLR 1:1; 5 min; 25°C) >> **F; SD** (3 h)	Local farms	Scallion?; *A. ascalonicum*; edible portion	(18)
gram-negative psychrotrophic bacteria		Log10 cfu/g<5 within 12 days	*Trachyspermum ammi*	**D** (50°C) >> E (EtOH 95%; SLR 1:10, 24 h) >> **F** >> **FD** (30°C)	Gorgan, Iran	shallot; *A. ascalonicum* L.; fruit	(31)
*Salmonella* sp.		**Frying**: 5.55 mm; **J:** 1.80 mm **dd** = 0 mm	–	**Frying** >> Water by-product **>>** **D** (r.t.); **J** (Water, SLR = 2:1) >> **E** (EtOH 98%, 3 days) >> **F** >> **FD**	Palu, Indonesia	Shallot; *A. ascalonicum;* Frying by-product	(25)
		**UAE**: 0.9 mm **dd** = 0 mm	*A. sativum*	**UAE** (Water; SLR = 1:10; 30°C; 15 min; 20 kHz) >> **C** (3,000 × g; 15 min; 4°C) >> **F**	BigC supermarket, Nha Trang, Vietnam	Shallot; *A. ascalonicum*; bulb	(19)
*Salmonella enterica*		**E:** 80;80;160 μL/ml		**E** (Water, UHT coconut milk, fresh coconut milk)	Bangkok, Thailand		(32)
*Salmonella Typhimurium*	ATCC 14028	**SD**: 5.3 ± 2.3; **PC:** 19.6 ± 1.3 mm **dd** = 6.0 mm	*A. sativum; A. cepa; A. porrum; A. tuberosum; A. schoenoprasum*	**SD (**turbo hydrodistillation;Water; SLR 1:1.6) >> +petroleum ether >> **F** >> **Ev** (40°C) (y = 80–120 mg/kg); 15 μl **PC:** 30 μg amoxicillin/ clavulanic acid	Avignon province, France	Shallot; *A. ascalonicum*; bulb	(21)
	*ATCC 13311*	**SD**: MIC = 2,400; MBC = 4,800	*##*	**D** (r.t., dark) >> **SD** (2 h) >> pH = 6; t = 8°C	West Azarbyjan, Iran	NA; *A. ascalonicum* L.; NA	(22)
	DT104	**B**: 7.67 ± 0.21; **E**: 6.24 ± 0.10; **SD 5** **μM**: 2.81 ± 0.10; **SD 10** **μM**: 1.44 ± 0.05; (log CFU/ml)	Shallot?; *(A. fistulosum)*	**E** (Water; SLR 1:1; 5 min; 25°C) >> **F; SD** (3 h)	Local farms	Scallion?; *A. ascalonicum*; edible portion	(18)
*Salmonella typhi*		**E**: MIC = 78.1 μg/ml	pH (4–11); 0–121°C; detergents; enzymes	**E** (Water; SLR 2:1; 5 h) >> **F** >> **D** (50°C) >> **Fract**: silica gel column (12–15/40)	NA	Shallot; *A. ascalonicum;* bulb	(17)
		**E**: MIC = 20,000; MBC = 20,000		**E** (Water; SLR 2:1, 5 h) >> **F**	Zagros mountains, Iran	Shallot/red onion; *A. ascalonicum;* bulb	(26)
	*ATCC19430*	**PC:** 13 mm; **J**: 10 mm; **Soxtec**: 12 mm; **dd** = 6 mm;	#	**J** (20 g); **Soxtec** **PC:** penicillin	Local market, Bangkok, Thailand	Shallot; *A. ascalonicum* L.; NA	(23)
*Salmonella para typhi A*		**E**: MIC = 10,000–20,000; MBC = 20,000		**E** (Water; SLR 2:1, 5 h) >> **F**	Zagros mountains, Iran	Shallot/red onion; *A. ascalonicum;* bulb	(26)
*Campylobacter jejuni*	ATCC 33291	**SD**: 5.6 ± 1.5 mm; **PC:** 19.3 ± 1.2 mm; **dd** = 6.0 mm	*A. sativum; A. cepa; A. porrum; A. tuberosum; A. schoenoprasum*	**SD (**turbo hydrodistillation;Water; SLR 1:1.6) >> +petroleum ether >> **F** >> **Ev** (40°C) (y = 80–120 mg/kg); 15 μl **PC:** 30 μg amoxicillin/ clavulanic acid	Avignon province, France	Shallot; *A. ascalonicum*; bulb	(21)
*Campylobacter* ssp.		**E**: MIC = 20,000; MBC = 20,000		**E** (Water; SLR 2:1, 5 h) >> **F**	Zagros mountains, Iran	Shallot/red onion; *A. ascalonicum;* bulb	(26)
*Shigella* sp.		**E**: MIC = 10,000–20,000		**E** (Water; SLR 2:1, 5 h) >> **F**	Zagros mountains, Iran	Shallot/red onion; *A. ascalonicum;* bulb	(26)
*Helicobacter pylori*	ATCC 24376	MIC = 6,250		**D** (sun) >> Soxhlet (MeOH, *n*-hexane; 24 h) >> **F** >> **FD**	Jos Market, Plateau State, Nigeria	shallot, multiplier onion; *A. ascalonicum*; leaves	(33)
	UCH 97001	MIC = 6,250		**D** (sun) >> Soxhlet (MeOH, *n*-hexane; 24 h) >> **F** >> **FD**	Jos Market, Plateau State, Nigeria	Shallot, multiplier onion; *A. ascalonicum*; leaves	(33)
	UCH 97009	MIC = 12,500		**D** (sun) >> Soxhlet (MeOH, *n*-hexane; 24 h) >> **F** >> **FD**	Jos Market, Plateau State, Nigeria	Shallot, multiplier onion; *A. ascalonicum*; leaves	(33)
	UCH 97009	MIC = 12,500		**D** (sun) >> Soxhlet (MeOH, *n*-hexane; 24 h) >> **F** >> **FD**	Jos Market, Plateau State, Nigeria	Shallot, multiplier onion; *A. ascalonicum*; leaves	(33)
	UCH 99039	MIC = 6,250		**D** (sun) >> Soxhlet (MeOH, *n*-hexane; 24 h) >> **F** >> **FD**	Jos Market, Plateau State, Nigeria	Shallot, multiplier onion; *A. ascalonicum*; leaves	(33)
*Acinetobacter baumannii*		**B**: 8.18 ± 0.23; **E**: 7.45 ± 0.20; **SD 5** **μM**: 3.71 ± 0.09; **SD 10** **μM**: 2.17 ± 0.05; (log CFU/ml)	Shallot?; *(A. fistulosum)*	**E** (Water; SLR 1:1; 5 min; 25°C) >> **F; SD** (3 h)	Local farms	Scallion?; *A. ascalonicum*; edible portion	(18)
**Antifungal activity**							
*Candida albicans*	ATCC 14053	**E**: MIC = 10,000,000		**E** (PBS; SLR 20:1; 30 min; r.t.) >> **C** (5,000 rpm; 10 min) >> **F** (0.22 μm) **PC:** clotrimazole (1.6 mg/ml?)	NA	Shallot; *A. ascalonicum;* NA	(34)
	ATCC 14053	**E**: MIC = 10–15		**D** (oven; 2 days) >> **E** (hexane; SLR 1:5) >> Soxhlet >> **F** >> **Ev**	NA	Persian Shallot; *A. ascalonicum* Linn.; fresh	(35)
	3092	**E:** MIC = 5–10		**D** (oven; 2 days) >> **E** (hexane; SLR 1:5) >> Soxhlet >> **F** >> **Ev**	NA	Persian Shallot; *A. ascalonicum* Linn.; fresh	(35)
		**J:** 25 mm **dd**: 10 mm		**J** (1%)	Ilam, Iran	Shallot; *A. ascalonicum* Linn.; Fresh bulbs	(36)
		**E:** IC_50_ = 96.00 ± 18.4 mg/ml	*Piper betle, Alpinia galanga*	**D** >> **E** (EtOH 95%; SLR 1:5; 24 h; 3 times; r.t.) >> **F** >> **Ev**	Nai Muang, Khon Kaen, Thailand	Shallot; *A. ascalonicum* L.; bulb	(37)
		**E**: MIC = 5,000; MFC =10,000		**E** (Water; SLR 2:1, 5 h) >> **F**	Zagros mountains, Iran	Shallot/red onion; *A. ascalonicum;* bulb	(26)
*Candida tropicalis*	ATCC 750	**E**: MIC = 20–30		**D** (oven; 2 days) >> **E** (hexane; SLR 1:5) >> Soxhlet >> **F** >> **Ev**	NA	Persian Shallot; *A. ascalonicum* Linn.; fresh	(35)
	5483	**E**: MIC = 10–20		**D** (oven; 2 days) >> **E** (hexane; SLR 1:5) >> Soxhlet >> **F** >> **Ev**	NA	Persian Shallot; *A. ascalonicum* Linn.; fresh	(35)
*Candida parapsilosis*	ATCC 22019	**E**: MIC = 10–20		**D** (oven; 2 days) >> **E** (hexane; SLR 1:5) >> Soxhlet >> **F** >> **Ev**	NA	Persian Shallot; *A. ascalonicum* Linn.; fresh	(35)
	2707	**E**: MIC = 10–20		**D** (oven; 2 days) >> **E** (hexane; SLR 1:5) >> Soxhlet >> **F** >> **Ev**	NA	Persian Shallot; *A. ascalonicum* Linn.; fresh	(35)
*Candida rugosa*	ATCC 10571	**E**: MIC = 20–30		**D** (oven; 2 days) >> **E** (hexane; SLR 1:5) >> Soxhlet >> **F** >> **Ev**	NA	Persian Shallot; *A. ascalonicum* Linn.; fresh	(35)
	3114	**E**: MIC = 20–30		**D** (oven; 2 days) >> **E** (hexane; SLR 1:5) >> Soxhlet >> **F** >> **Ev**	NA	Persian Shallot; *A. ascalonicum* Linn.; fresh	(35)
*Candida krusei*	ATCC 6258	**E**: MIC = 30–40		**D** (oven; 2 days) >> **E** (hexane; SLR 1:5) >> Soxhlet >> **F** >> **Ev**	NA	Persian Shallot; *A. ascalonicum* Linn.; fresh	(35)
	3109	**E**: MIC = 30–40		**D** (oven; 2 days) >> **E** (hexane; SLR 1:5) >> Soxhlet >> **F** >> **Ev**	NA	Persian Shallot; *A. ascalonicum* Linn.; fresh	(35)
*Saccharomyces cerevisiae*		**E**: MIC = NE MMC = NE; **PC:** MIC = 0.312; MMC = 0.312	**-**	**E** (EtOH 95%; SLR 1:1; one night, r.t.) >> **Ev** >> **FD** **PC:** amphotericin B	Thai market, Pathumthani, Thailand	Shallot; *A. ascalonicum* Linn.; bulb	(20)
		**E**: MIC = 1,200; MFC = 2,500		**E** (Water; SLR 2:1, 5 h) >> **F**	Zagros mountains, Iran	Shallot/red onion; *A. ascalonicum;* bulb	(26)
*Syncephalastrum* sp.		**J:** 22 mm **dd**: 10 mm		**J** (1%)	Ilam, Iran	Shallot; *A. ascalonicum* Linn.; Fresh bulbs	(36)
*Penicillium* sp.		**J:** 28.7 mm **dd**: 10 mm		**J** (1%)	Ilam, Iran	Shallot; *A. ascalonicum* Linn.; Fresh bulbs	(36)
*Paecilomyces* sp.		**J**: 22.7 mm **dd**: 10 mm		**J** (1%)	Ilam, Iran	Shallot; *A. ascalonicum* Linn.; Fresh bulbs	(36)
*Aspergillus niger*		**J:** 22 mm **dd**: 10 mm		**J** (1%)	Ilam, Iran	Shallot; *A. ascalonicum* Linn.; Fresh bulbs	(36)
		**E**: MIC = 62.5 μg/ml	pH (4–11); 0–121°C; detergents; enzymes	**E** (Water; SLR 2:1; 5 h) >> **F** >> **D** (50°C) >> **Fract**: silica gel column (12–15/40)	NA	Shallot; *A. ascalonicum;* bulb	(17)
		**E**: MIC = 10,000–20,000;MBC = 20,000		**E** (Water; SLR 2:1, 5 h) >> **F**	Zagros mountains, Iran	Shallot/red onion; *A. ascalonicum;* bulb	(26)
	CCRC 32126	**E:** MFC = 228 μg/ml	*A. sativum; A. fistulosum; A. bakeri; A. odorum; A. tuberosum; A. cepa*	**E** (Water; SLR 1:1; 3 min; 25°C) >> **F**	Local markets	Shallot; *A. ascalonicum* L; bulb	(38)
*Aspergillus flavus*		**E**: MIC = 20,000		**E** (Water; SLR 2:1, 5 h) >> **F**	Zagros mountains, Iran	Shallot/red onion; *A. ascalonicum;* bulb	(26)
	CCRC 30146	**E**: MFC = 558 μg/ml	*A. sativum; A. fistulosum; A. bakeri; A. odorum; A. tuberosum; A. cepa*	**E** (Water; SLR 1:1; 3 min; 25°C) >> **F**	Local markets	Shallot; *A. ascalonicum* L; bulb	(38)
	MTCC 10680	**E**:49.7% inhibition at 2500 μL	*A. cepa; A. sativum*	**E** (Water; SLR 1:1; 24 h; 4°C) >> **F** >> **Ev**	Tiruchengode, Tamil Nadu, India	Shallot; *A. cepa* var. *aggregatum;* bulb	(39)
*Aspergillus fumigatus*		**E**: MIC = 20,000		**E** (Water; SLR 2:1, 5 h) >> **F**	Zagros mountains, Iran	Shallot/red onion; *A. ascalonicum;* bulb	(26)
	CCRC 3052	**E**: MFC = 474 μg/ml	*A. sativum; A. fistulosum; A. bakeri; A. odorum; A. tuberosum; A. cepa*	**E** (Water; SLR 1:1; 3 min; 25°C) >> **F**	Local markets	Shallot; *A. ascalonicum* L; bulb.	(38)
*Cladosporium* sp.		**J**: 29 mm **dd**: 10 mm		**J** (1%)	Ilam, Iran	Shallot; *A. ascalonicum* Linn.; Fresh bulbs	(36)
*Alternaria* sp.		**J**: 22 mm **dd**: 10 mm		**J** (1%)	Ilam, Iran	Shallot; *A. ascalonicum* Linn.; Fresh bulbs	(36)
*Drechslera* sp.		**J**: 38 mm **dd**: 10 mm		**J** (1%)	Ilam, Iran	Shallot; *A. ascalonicum* Linn.; Fresh bulbs	(36)
*Microsporum canis*		**E:** IC_50_ = 69.87 ± 45.5 mg/ml	*Piper betle, Alpinia galanga*	**D** >> **E** (EtOH 95%; SLR 1:5; 24 h; 3 times; r.t.) >> **F** >> **Ev**	Nai Muang, Khon Kaen, Thailand	Shallot; *A. ascalonicum* L.; bulb	(37)
*Microsporum gypseum*		**J**: 29 mm **dd**: 10 mm		**J** (1%)	Ilam, Iran	Shallot; *A. ascalonicum* Linn.; Fresh bulbs	(36)
		**E:** IC_50_ = 73.33 ± 22.6 mg/ml	*Piper betle, Alpinia galanga*	**D** >> **E** (EtOH 95%; SLR 1:5; 24 h; 3 times; r.t.) >> **F** >> **Ev**	Nai Muang, Khon Kaen, Thailand	Shallot; *A. ascalonicum* L.; bulb	(37)
		**E**: MIC = 150; MFC = 2,500		**E** (Water; SLR 2:1, 5 h) >> **F**	Zagros mountains, Iran	Shallot/red onion; *A. ascalonicum;* bulb	(26)
*Trichophyton mentagrophytes*		**E**: 25.7–28.3 mm **dd**: 10 mm		**J** (1%)	Ilam, Iran	Shallot; *A. ascalonicum* Linn.; Fresh bulbs	(36)
		IC_50_ = 61.20 ± 15.0 mg/ml	*Piper betle, Alpinia galanga*	**D** >> **E** (EtOH 95%; SLR 1:5; 24 h; 3 times; r.t.) >> **F** >> **Ev**	Nai Muang, Khon Kaen, Thailand	Shallot; *A. ascalonicum* L.; bulb	(37)
		**E**: MIC = 620; MFC = 2,500		**E** (Water; SLR 2:1, 5 h) >> **F**	Zagros mountains, Iran	Shallot/red onion; *A. ascalonicum;* bulb	(26)
*Trichophyton rubrum*		**E**: MIC *=* 156.2	pH (4–11); 0–121°C; detergents; enzymes	**E** (Water; SLR 2:1; 5 h) >> **F** >> **D** (50°C) >> **Fract**: silica gel column (12–15/40)	NA	Shallot; *A. ascalonicum;* bulb	(17)
		**E**: MIC = 1,200–2,500; MFC = 5,000		**E** (Water; SLR 2:1, 5 h) >> **F**	Zagros mountains, Iran	Shallot/red onion; *A. ascalonicum;* bulb	(26)
*Epidermophyton floccosum*		**J**: 26–30 mm **dd**: 10 mm		**J** (1%)	Ilam, Iran	Shallot; *A. ascalonicum* Linn.; Fresh bulbs	(36)
*Cryptococcus humicolus*		**E**: MIC = 156.2	pH (4–11); 0–121°C; detergents; enzymes	**E** (Water; SLR 2:1; 5 h) >> **F** >> **D** (50°C) >> **Fract**: silica gel column (12–15/40)	NA	Shallot; *A. ascalonicum;* bulb	(17)
		**E**: MIC = 2,500–5,000; MFC = 10,000		**E** (Water; SLR 2:1, 5 h) >> **F**	Zagros mountains, Iran	Shallot/red onion; *A. ascalonicum;* bulb	(26)
*Aureobasidium pullulans*		**E**: MIC = 150–310; MFC = 5,000		**E** (Water; SLR 2:1, 5 h) >> **F**	Zagros mountains, Iran	Shallot/red onion; *A. ascalonicum;* bulb	(26)
*Fusarium oxysporum*		**E**: MIC = 1,200–2,500; MFC = 10,000		**E** (Water; SLR 2:1, 5 h) >> **F**	Zagros mountains, Iran	Shallot/red onion; *A. ascalonicum;* bulb	(26)
**Antiprotozoal and antiviral activity**							
*Sitophilus; zeamais*		Repellency; Larvae: 63.75%; Adults: 66.25% Toxicity; Larvae: 50.29%; Adults: 26.35%	*Mentha haplocalyx*	**D** (oven; 3 days) >> **F** >> **E1** (EtOH 95%; SLR 1:5; 7 days; 25°C) >> **F** >> **E2** (EtOH 95%; SLR 1:2.5; 7 days; 25°C); **E1**+**E2** >> **Ev**	Beijing; Tongrentang Group, China	NA; *A. ascalonicum;* NA	(40)
*Leptotrombidium deliense*		Repellency rate: 30%; 53%; 56%; 73%; 86%; 93%		**J** >> **F** >> **C** (5,000 rpm) 0.01%; 0.1%; 1%; 2.5%; 5%; 10%	NA	Onion; *A. cepa* var. *aggregatum;* NA	(41)
*Adenovirus ADV41* and *ADV3*		**ADV41**: SI = 3.7 (2,696.8/733.9); **ADV3**: SI = 2.4 (2,696.8/1,137.6)	*A. porrum; A. sativum; A. cepa; A. fistulosum*	**D** (air; dark; 22°C) >> **FD** (24 h) >> **E** (Water)	Yun-Lin, Taiwan	Shallot; *A. ascalonicum* L.; NA	(42)

*All MIC and MFC values are expressed in μg/ml; B, blank; negative control; E, extraction; D, drying; SD, steam distillation; F, filtered; FD, freeze-drying; Ev, evaporation; Fract, fractionation; C, centrifugation; PC, positive control; UAE, ultrasound-assisted extraction; Soxhlet, Soxhlet extraction; IRR, irradiation; J, juice; dd, disk diameter; SLR, solid-liquid ratio (mg/ml)*.

# *Coriandrum sativum; Zingiber offcinale; Alpinia galanga; Cymbopogon citratus; Citrus hystrix; Citrus aurantiifolia; Capsicum frutescens; Curcuma longa; Ocimum basilicum; Ocimum sanctum; Momordica charantia; Solanum torvum; Morinda citrifolia*.

## *Mentha longifolia; Cuminum cyminum; Zataria multiflora; Pimpinella anisum*.

#### Gram-Positive Bacteria Inhibitory Activity

**Figure 2:**
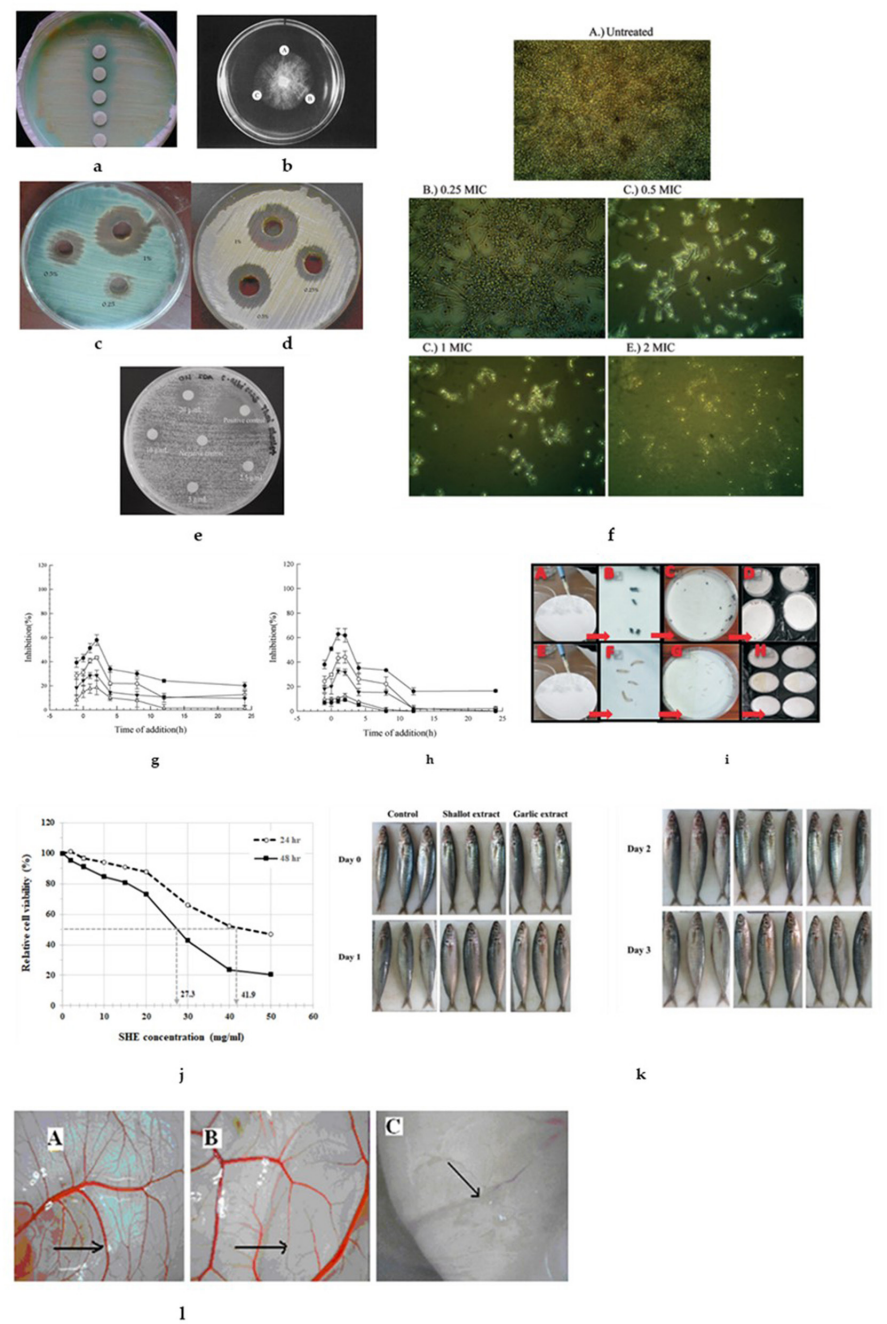
Antimicrobial effects of shallot. **(a)** Antimycobacterial activity of partially purified extract of *Allium ascalonicum*. **(b)** Anti-fungal activity of ascalin against *B. cinerea*. (A) Control, (B) Ascalin (75 μg), and (C) Ascalin (15 μg). Shallot extract effect on **(c)** *Penicillium* sp., **(d)** *Candida albicans*, **(e)** *Allium ascalonicum* aqueous extracts inhibited the growth of *Candida albicans*. **(f)** Biofilm formation observed after 24 h with shallot extract (A) without treatment (B) 0.25 MIC, (C) 0.5 MIC, (D) 1 MIC and (E) 2 MIC at 40× magnification. A reduction of activity was observed with increasing concentration of treatment. Inhibitory effect of adding shallot at various times pre-infection or post-infection of adenovirus [ADV-41 **(g)**; ADV-3 **(h)**] to A549 cells. Different concentrations of shallot [120 mg/L (open triangles), 240 mg/L (filled triangle), 480 mg/L (open circles), 960 mg/L (filled circles)], were added at various times pre-infection (~1 h), co-infection (0 h) or post-infection (1–24 h) of adenovirus (ADV-41; ADV-3) to A549 cells at 37°C. The x-axis indicates the time course of adding shallot (42). **(i)** Adult and larvae maize weevil fumigant toxicity experiment: (A) application of essential oil on filter paper (B) filter paper in jar lid; (C) maize weevil adults; (D) fumigant toxicity effects observed in the jar; (E) jars maintained at rearing temperature and humidity; (F) application of essential oil on filter paper; (G) filter paper in jar lid; (H) maize weevil larvae; (I) fumigant toxicity effects observed in the jar; (J) jars maintained at rearing temperature and humidity (29, 34, 36, 40, 45). **(j)** The growth-inhibitory effect of shallot juice extract in EA.hy926 cells. **(k)** Apparent changes occurred in round scad during ice storage. Shallot extract, the round scad was immersed in shallot extract; Garlic extract, the round scad was immersed in garlic extract; Control, the round scad was immersed in distilled water (19, 46). **(l)** Anti-angiogenic effect of ethyl acetate fraction on CAM model of angiogenesis (arrows show neovascular forming in the chicken chorioallantoic membrane); (A) control, (B) treatment with EA fraction at 3 ng/egg, (C) treatment with ethyl acetate fraction at 10 ng/egg (47).

Bowles and Juneja (43) demonstrated that ethanolic shallot extract inhibited bacterial growth of *Clostridium bolutinum* spores cultured in a medium enriched with brain heart infusion broth. In addition, Saenthaweesuk et al. demonstrated the potential of shallot to inhibit gram-positive bacteria *Staphylococcus epidermidis* and *Bacillus subtilis* ATCC 6633 with a minimum inhibitory concentration (MIC) and minimum microbicidal concentration (MMC) varying in the range of 25–50 and 25–200 mg/ml, respectively. However, compared to control (gentamycin), the inhibitory activity exerted by these extracts is very weak (~5–6 orders of magnitude lower). A study conducted by Adeleye et al. (44) revealed that both water and ethanolic extracts of shallot were efficient in inhibiting the proliferation of *Mycobacterium tuberculosis*. Similarly, Mansour et al. (29) documented a strong inhibition potential on *M. tuberculosis*, as is shown in Figure 2a. However, these studies used no positive control, thus making the results barely applicable.

#### Gram-Negative Bacteria Inhibitory Activity

Methanolic shallot extract demonstrated a detectable inhibitory potential against three of the non-tuberculous mycobacteria species (*Mycobacterium fortuitum, M. smegmatis*, and *M. phlei*) with a MIC of 100 mg/ml, and 72–98% kill of susceptible organisms in 24 h (27). However, the inhibition activity exerted by these extracts is very weak in comparison with standard rifampicin (MIC = 40 μg/ml). Conversely, a study that aimed to compare the antibacterial effect of three common *Allium* species (garlic, shallot, and onion) found that among tested methanolic extracts, shallot exerted the lowest inhibition capacity against *Klebsiella* spp. and *Micrococcus* spp. strains (30). These results are similar to those reported by Maidment et al. (48), where ethanolic shallot extract yielded no inhibitory effect on *E. coli* strains when compared to 12 other *Allium* species. However, Nemati et al. (16) revealed that fresh lyophilized powder of shallot is effective against *E. coli, Klebsiella*, and *Staphylococcus aureus* (with a MIC of 3.29, 3.6, and 3.8 mg/ml, respectively). These results are further confirmed by a more recent study by Danquah et al. (28), who reported that Ethanol and aqueous extracts of shallot inhibited the growth of *Mycobacterium smegmatis* and *Escherichia coli*.

Adeniyi and Anyiam (33) reported that shallot extract was effective against five strains of *Helicobacter pylori*, with MIC ranging from 6.25 to 12.5 mg/ml. They concluded that increasing the concentration of the extract decreased the urease activity of all tested strains.

#### The Antibacterial Effect of Shallot Essential Oil

Shallot essential oil significantly inhibited the growth of 4 food-borne bacteria (*Salmonella typhimurium* DT104, *E. coli* O157:H7, *Listeria monocytogenes*, and *Staphylococcus aureus*) and 4 nosocomial bacteria (MRSA, *Klebsiella pneumoniae, Pseudomonas aeruginosa*, and *Acinetobacter baumannii*) (18). However, in an attempt to compare the antimicrobial effect exerted by this species with the antimicrobial effect of *A. fistulosum*, the authors mixed up the popular names of the species, making the results unreliable. Mahmoudi et al. (22) reported a MIC of 150 μg/ml against *S. aureus* at 8°C, pH 6. Mnayer et al. (21) reported that shallot essential oil was extremely effective on *S. aureus* (20.0 mm) and active on *Campylobacter jejuni* (11.6 mm) and *Salmonella typhimurium* (11.3 mm). Moreover, an additional study reported that shallot essential oil (2,000 ppm) can act as a preservative in foods, decreasing the mean bacterial colonies of *E. coli* during the ripening period of Iranian White Brined Cheese, without interfering with the organoleptic properties (49). Based on this study, the group of Silva et al. confirmed, using a meta-regression model, that the shallot essential oil can inhibit the growth of *E. coli* strains, similar to lemon balm, sage, and anise essential oils (50).

#### The Antibacterial Effect of Isolated Compounds From Shallot

Recently, to characterize the antimicrobial agents from shallot, van Chuyen et al. (51) isolated and assessed 5 terpenoid compounds. The results showed that isolated saponins (i.e., 3,24-acetonideclethric acid, ursolic acid, randiasaponin IV, lekudinoside W, and (25S)-1β,3β,24β-trihydroxyspirost-5-en 1-*O*-α-L-rhamnopyranosyl-(1 → 2)-α-L-arabinopyranoside) can inhibit the proliferation of *E.coli, S. aureus* as well as *C. albicans* with IC_50_ ranging from 36.62 to 101.99 μM.

#### Preservation Proprieties of Shallot

In a study assessing 15 Thai food ingredients and their antimicrobial activity, ethanolic shallot extract inhibited the proliferation of several bacterial strains such as *Bacillus cereus, Salmonella typhi*, and *Staphylococcus aureus* (23). However, even if control compounds were tested (deoxytetracycline, penicillin, and gentamycin, respectively), their dosage is not mentioned in the methodology. As a result, the applicability of this study is scarce. Similarly, Amin and Kapadnis showed that dried and autoclaved extracts of shallot showed higher inhibitory activity on *Bacillus cereus and Microsporum gypseum* than similar extracts of onion and garlic (26). Furthermore, Mekvimo et al. (14) assessed the antimicrobial activity of three Thai herbs against *S. agalactiae*, showing that the ethanolic crude extract of red shallot has the most potent activity followed by mulberry leaves and marigold petal extract. Thus, considering the effectiveness of spices and herbs as natural sources of antimicrobial compounds, Raeisi et al. (31) evaluated the shallot inhibitory effect on gram-negative psychrotrophic bacteria, an important group of microorganisms that are well known as the main cause of aerobic spoilage of stored fish at low temperatures, concluding that shallot extract could be utilized as a safe preservation method for extending the shelf life of fish products due to its inhibition of microbial growth, delayment of chemical deterioration, and maintenance or improvement of sensory attributes. In a similar study, Duy Bao et al. concluded that the aqueous extract of shallot can extend the shelf-life of round scad (*Decapterus maruadsi*) up to 4 weeks under ice storage conditions. They also revealed that this extract can inhibit the proliferation of several Gram-positive (i.e., *Bacillus cereus, Staphylococcus aureus*) and Gram-negative (i.e., *Escherichia coli, Salmonella typhimurium*) bacterial strains, however, with a lower inhibitory effect compared to garlic (19). Contrary to these results, Abdollahzadeh et al. (52) revealed that the aqueous extract of shallot has no significant effect in extending the shelf-life of silver carp (*Hypophthalmichthys molitrix*) inoculated with *L. monocytogenes* PTCC 1163 strain at 4 ± 1°C.

#### Gut Microbiota

Knowing the beneficial effects of the balance between non-pathogenic and pathogenic bacteria, Mozin et al. (25) conducted a study demonstrating that shallot by-products (dry skin, outer and inner layers, and top-bottom of the shallot) inhibited the growth of *E. coli* and *Salmonella* spp. whereas tended to increase the growth of *Lactobacillus* spp. bacteria in the gastrointestinal tract of chickens. The authors ascribe this effect partially to quercetin, a compound found in these by-products. However, this observation is not supported by any other studies. A recent review concerning the effect of quercetin on gut microbiota highlights that quercetin is transformed by the microbiota into 3,4-dihydroxyphenylacetic acid, also known as homoprocatechuic acid, 3-(3-hydroxyphenyl)propionic acid, 3,4-dihydroxybenzoic acid and 4-hydroxybenzoic acid. *B. fragilis, C. perfringens, E. ramulus, Streptococcus* S-2, *Lactobacillus* L-2, *Bifidobacterium* B-9 and *Bacteroides* JY-6 have been identified as the bacterial strains responsible for the transformation of quercetin into the metabolites mentioned above (53).

#### The Influence of Thermally and Non-thermally Processes on Shallot's Antibacterial Activity

In terms of compounds' stability, Yasurin and Saenghiruna assessed the antibacterial activity of shallot extracted in water, UHT coconut milk, and fresh coconut milk as people use to cook in Thai traditional cuisine. The study revealed that shallot compounds are stable under Thai traditional cooking conditions (1 h of boiling at 98–100°C) and have the potential to inhibit *S. enterica* (MIC of 80, 80, and 160 μg/ml for water, UHT coconut milk, and fresh coconut milk extracts, respectively) (32). These results are confirmed by the study of Mansour et al. (17), which concluded that antimicrobial activity of shallot water extract is maintained under different extraction conditions (pH, temperature, the addition of proteolytic enzymes and surfactants), revealing that the shallot inhibition capacity is not influenced by these external factors, its chemical composition being stable. However, the authors did not evaluate the chemical composition for shallot extracts treated in given conditions, nor did they use a positive control for evaluating the inhibitory activity exerted on microbial strains, thus the applicability of these results is scarce.

The group of Sinuraya et al. (24) studied the mutagenic effect of gamma irradiation on the antimicrobial potential of shallot. Their study revealed that the ethanolic extract of shallot samples irradiated at 2 and 6 Gy inhibited the proliferation of *S. aureus* (MIC = 512 μg/ml).

### Antifungal Activity

In a study in which researchers attempted to investigate the antifungal capacity of *A. ascalonicum* was discovered that shallot bulbs contain an anti-fungal peptide (see next section Antiviral Activity for more details), designated as ascalin. They used a complex isolation procedure to separate it, involving ion-exchange chromatography on DEAE–cellulose, affinity chromatography on Affi-gel blue gel, ion-exchange chromatography on SP–Sepharose, and gel filtration on Superdex 75. Further characterization showed that ascalin has a molecular weight of 9.5 kDa and a YQCGQGG N-terminal sequence, which is similar to chitinase from other *Allium* species, however, the latter has a greater molecular weight (45). Ascalin inhibited mycelial growth in the fungus *Botrytis cinerea* (see Figure 2b), but not in the fungi *Mycosphaerella arachidicola* and *Fusarium oxysporum* (45).

#### Zoonotic Dermatophytes Inhibition

Crude extracts of *A. ascalonicum* bulbs tested against selected zoonotic dermatophytes (*Microsporum canis, Microsporum gypseum*, and *Trichophyton mentagrophyte*) and *Candida albicans* strains showed a good inhibitory activity with MIC ranging between 61.20–96.00 μg/ml (37). These findings were confirmed by further research where shallot extracted by agar well diffusion method exhibited remarkable activity against saprophytic fungi, followed by *Candida* species and dermatophytes, as depicted in Figures 2c,d (36).

#### Antifungal Potential on *Candida* spp.

In a recent study, hexane shallot extract exerted high potency in inhibiting *Candida* spp. strains (MIC ranging 5–50 μg/ml, for different species of *Candida*). In contrast, the aqueous extract was able to inhibit the growth of *Candida albicans* only at a higher concentration of 10 g/ml (Figure 2e) (34). This effect can be ascribed to the different polarity of phytochemicals extracted in aqueous and ethanolic media, respectively (54).

However, ethanolic and even aqueous shallot extract inhibited *Candida* biofilm formation. The mechanism involved in the inhibition of *Candida* biofilm formation is thought to be the down-regulation of the *HWP1* gene (34, 35). Figure 2f shows the *Candida* biofilm formation and its inhibition in presence of shallot extract.

#### The Influence of Thermal Processes on Shallot's Antifungal Activity

The shallot extract inhibited the growth of three *Aspergillus* species with an MFC ranging from 228 to 558 μg/ml. The inhibitory activity decreased with increasing incubation and heating temperature, whereas the pH adjustment with acetic acid increased this effect. Interestingly, the extract processed in a medium of a pH of 4 followed by the application of heat treatment exerted the highest activity against *Aspergillus* species (38). A more recent study evaluated the susceptibility of Groundnut oil isolate *Aspergillus flavus* MTCC 10680 to several *Allium* extracts (39). It was found that shallot extracts exhibited concentration-dependent *in vitro* inhibition on mycelial biomass, radial growth, and toxin elaboration, proving that culinary shallot oil seasoning has anti-aflatoxin and food additive potential for use in food industries (55).

A summary of all antifungal proprieties of shallot extract is provided in Table 1.

### Antiviral Activity

Employing an MTT assay with human lung carcinoma cells (A549), Chen et al. (42) documented that among five *Allium* species, the extract from shallot exerted the highest antiviral activity against two adenovirus strains (ADV41 and ADV3), especially during the first 2 h of infection (during the early period of virus replication). Figures 2g,h reveals the inhibitory effect of shallot effect on ADV-41 and ADV-3 infected A539 cell lines.

Besides its antifungal activity, ascalin has been found to possess anti-viral activity as well. Ascalin inhibited the HIV-1 reverse transcriptase with an IC_50_ of 10 μM (45).

### Antiprotozoal/Antiparasitic

Antiprotozoal and antiparasitic effects of shallot have been documented in a few studies, which are summarized in Table 1, along with studies related to antiviral activity.

Azadbakht et al. (56) reported that a 0.2 mg/ml shallot extract can substantially inhibit *Giardia* proliferation. Another study concluded that ethanol-extracted essential oil of *A. ascalonicum* has repellent and toxic effects against adults and larvae of maize weevil, *Sitophilus zeamais*. Compared to the extract of *Mentha haplocalyx*, shallot extract showed a better toxic effect on larvae, which is illustrated in Figure 2i (40).

Furthermore, one study indicated that shallot extract could have potential use as a symbiotic agent. Chalorsuntisakul et al. aimed to investigate the influence of a symbiotic formula consisting of 10^6^ CFU/ml of *Lactobacillus plantalum* CMUFP002, and 2% shallot extract *v*/*v* mix in water on *Eimeria tenella* infections in broiler chickens. They concluded that this symbiotic formula may have a preventative effect on coccidiosis in broilers, but do not fully protect against the negative impact of the infection (57). Another study revealed that among 7 crude plant extracts, 0.01% shallot exhibited the highest repellency activity on larval *Leptotrombidium delicense* (30%) (41).

## Antioxidant and Anti-Inflammatory Activity

Non-communicable chronic diseases (NCD) raise public health concerns, bringing into attention of authorities a different type of pandemic (58). One of the pathogeneses of NCD is strongly correlated to oxidative stress, which is caused by an imbalance in the production of the reactive oxygen species (ROS) and the ability of the endogenous antioxidant system to neutralize these species. The mechanism of oxidative stress implies tissue and cell damage, such as endothelial cell damage, with the installation and acceleration of the inflammation process, thus triggering endothelial dysfunction, factors affecting the initiation and progression of atherosclerosis (59). In recent years, plant extracts with anti-oxidative and anti-inflammatory properties, or compounds that show protective properties against damages induced by ROS, are under intense research (46). The main findings regarding both anti-inflammatory and antioxidant activity of shallots are discussed below, and a summary of main findings regarding the antioxidant activity of shallot extracts can be found in Table 2.

**Table 2:** Summary of main antioxidant activity exerted by shallot extracts.

**Method**	**Results**	**Extraction procedure**	**Zone**	**Species**	**References**
TPC	**E**: 1658.17 mg GAE/g fw	**J** **>>** **F** (sterile gauze) >> **C** (3,000 rpm; 4°C; 15 min) >> **F** (Whatman no.1) >> **FD**	Chiangmai, Thailand	**BN**: *A. ascalonicum* cv. *Chiangmai* **P**: bulb	(46)
	**THD**: 11.14 mg GAE/g fw **PC**: 46.77 mg GAE/g fw	**THD** (Water; 1:1.6) >> +petroleum ether >> **F** (Na_2_SO_4_) >> **Ev** (40°C) **PC**: BHT	Avignon, France	**PN**: shallot **BN**: *A. ascalonicum* **P**: bulb	(21)
	**E**: 1.30 mg GAE/g fw	**E** (Water; 1:2)	Mendoza, Argentina	**PN**: shallot **BN**: NA **P**: bulb	(60)
	**E**: 2.5 mg GAE/g dw	**D** (50°C) >> powdered (80 mesh) >> **E** (apple juice; 1:48; 96°C; 30 min)	Ubon Ratchathani, Thailand	**PN**: shallot **BN**: *A. ascalonicum* L. **P**: bulb	(61)
	**E**: 9.59–10.31 mg GAE/g dw	**E** (chlorine solution; 3:1; 1 min) >> **F** (sieve; 60 min) >> 38% (*w*/*w*) in Keang-hleung paste	Hatyai, Songkhla province, Thailand	**PN**: shallot **BN**: *A. ascalonicum* L. **P**: bulb	(62)
	**E** (ethyl acetate): 400 mg GAE/g dw	**D** (40°C; 3 days) >> **E** (n-hexane ×3 >> ethyl acetate ×3 >> EtOH ×3)	Caringin market, Bandung, Indonesia	**PN**: red onion **BN**: *A. cepa* L. var. *ascalonicum* Backer **P**: bulb	(63)
	**E**: 0.06 mg GAE/g dw	**E** (Water; 1:2; 12 h) >> **F** (cheesecloth) >> **C** (4,000 × g for 30 min) >> **Ev** (50°C)	Hat Yai, Songkhla, Thailand	**PN**: shallot **BN**: *A. ascalonicum* L. **P**: bulbs	(64)
	**UAE**: 8.82 ± 0.06 μg GAE/ml extract	**UAE** (Water; 1:10; 15 min at 30°C, 20 kHz) >> **C** (3.000 × *g*; 15 min; 4°C)	BigC supermarket, Nha Trang, Vietnam	**PN**: shallot **BN**: *A. ascalonicum* **P**: bulb	(19)
	**E**: 0.98 mg PE/g	**E** (MeOH; 1:2; 4°C; 11 min) >> **F** (cheesecloth) >> **E** >> **Ev** (65°C)	NA	**PN**: Shallot **BN**: *A. ascalonicum* **P**: bulb	(30)
	**Snack**: 162.32 mg GAE/L	**D (**40°C; 96 h) >> **M** (60 mm mesh sieve) >> snack (25%)	Shasha market, Akure, Ondo State, Nigeria	**PN**: Shallot **BN**: *A. ascalonicum* **P**: bulb	(65)
TFC	**E**: 0.098 mg QE/g fw	**J** **>>** **F** (sterile gauze) >> **C** (3000 rpm; 4°C; 15 min) >> **F** (Whatman no.1) >> **FD**	Chiangmai, Thailand	**BN**: *A. ascalonicum* cv. *Chiangmai* **P**: bulb	(46)
	**E**: ~4 mg QE/g dw	**D** (50°C) >> powdered (80 mesh) >> **E** (apple juice; 1:48; 96°C; 30 min)	Ubon Ratchathani, Thailand	**PN**: shallot **BN**: *A. ascalonicum* L. **P**: bulb	(61)
	**E** (ethyl acetate): 89.30 mg QE/g extract	**D** (40°C; 3 days) >> **E** (n-hexane ×3 >> ethyl acetate ×3 >> EtOH ×3)	Caringin market, Bandung, Indonesia	**PN**: red onion **BN**: *A. cepa* L. var. *ascalonicum* Backer **P**: bulb	(63)
	**J**: 0.09 mg CE/g fw	**J** (Water; minimum amount) >> **F** >> **C** (12000 × g; 20 min; 4°C) >> **FD**	Hsinchu, Taiwan	**PN**: Shallot **BN**: *A. ascalonicum* **P**: bulb	(66)
	**E**: 0.06 mg GAE/g sample	**E** (Water; 1:2; 12 h) >> **F** (cheesecloth) >> **C** (4,000 × g for 30 min) >> **Ev** (50°C)	Hat Yai, Songkhla, Thailand	**PN**: shallot **BN**: *A. ascalonicum* L. **P**: bulb	(64)
	**E**: 1.42 mg/g fw	**E** (Water; 1:2)	Mendoza, Argentina	**PN**: shallot **P**: bulb	(60)
	**Snack**: 86.70 mg QE/L	**D (**40°C; 96 h) >> **M** (60 mm mesh sieve) >> snack (25%)	Shasha market, Akure, Ondo State, Nigeria	**PN**: Shallot **BN**: *A. ascalonicum* **P**: bulb	(65)
DPPH	**E** (MAC): IC_50_ = 131.69 μg/ml **E** (PER): IC_50_ = 55.62 μg/ml **E** (REF): IC_50_ = 31.31 μg/ml **E** (SOX): IC_50_ = 126.05 μg/ml	**E** (EtOH 70% + HCl 2N; 1:10; pH 1; MAC: 24 h; PER: 8 h; SOX: 2 h; REF: 2 h) ×3 times >> **Ev** (40°C) >> **Fract** (complex)	Gede Bage market, West Java, Indonesia	**PN**: Maja Cipanas Onion **BN**: *A. cepa* L. var. *ascalonicum* **P**: skin	(67)
	**THD**: IC_50_ = 2700 μg/ml **PC**: IC_50_ = 30 μg/ml	**THD** (Water; 1:1.6) >> +petroleum ether >> **F** (Na_2_SO_4_) >> **Ev** (40°C) **PC**: BHT	Avignon, France	**PN**: shallot **BN**: *A. ascalonicum* **P**: bulb	(21)
	**E**: IC_50_ = 3710 μg/ml	**J** (Water; minimum amount) >> **F** >> **C** (12000 × *g*; 20 min; 4°C) >> **FD**	Hsinchu, Taiwan	**PN**: shallot **BN**: *A. ascalonicum* **P**: bulb	(66)
	**E**: IC_50_ >437.375 μg/ml	**E** (EtOH 80%; 1:30; 200 rpm; 24 h; r.t) >> **F** >> **E** (40°C)	Samosir, Indonesia	**BN**: *A. cepa* L. var. *ascalonicum* Backer **P**: bulbs	(24)
	**E**: 1.30–2.03 mg GAE/g dw	**E** (chlorine solution; 3:1; 1 min) >> **F** (sieve; 60 min) >> 38% (*w*/*w*) in Keang-hleung paste	Hatyai, Songkhla province, Thailand	**PN**: shallot **BN**: *A. ascalonicum* L. **P**: bulb	(62)
	**E**: 1.73–2.36 mg TE/g dw	**E** [MeOH; 1:20; modified SOX (Büchi B-811 extraction system)] >> **Ev**	Bejo Zaden, Holland	**PN**: shallot **BN**: *A. cepa* var. *ascalonicum* Backer: cv. Conservor F1, Bonilla F1 and Matador F1 **P**: bulb	(68)
	**E**: ~1.37 mg TE/g dw	**FD** (5 days) >> **M** (1 mm sieve) >> **E** (EtOH 70%; 1:20; 2 h; 300 rpm) >> **C** (4500 rpm; 10 min)	Tabernal-Zaden, Venhuizen, The Netherlands	**PN**: shallot **BN**: *A. ascalonicum*): cv. ‘Creation' F1 **P**: white part	(69)
	**E**: 11.81 mg GAE/g fw	**E** (Water; 1:2; 12 h) >> **F** (cheesecloth) >> **C** (4,000 x g for 30 min) >> **Ev** (50°C)	Hat Yai, Songkhla, Thailand	**PN**: shallot **BN**: *A. ascalonicum* L. **P**: bulb	(64)
	**UAE**: 0.5 ml extract: ~0.35 mM **PC**: 0.5 mg BHT: ~0.4 mg mM	**UAE** (Water; 1:10; 15 min at 30°C, 20 kHz) >> **C** (3.000 × *g*; 15 min; 4°C) **PC**: BHT	BigC supermarket, Nha Trang, Vietnam	**PN**: shallot **BN**: *A. ascalonicum* **P**: bulb	(19)
	**E** (ethyl acetate): 40.22 ± 3,06%	**D** (40°C; 3 days) >> **E** (n-hexane ×3 >> ethyl acetate ×3 >> EtOH ×3)	Caringin market, Bandung, Indonesia	**PN**: red onion **BN**: *A. cepa* L. var. *ascalonicum* Backer **P**: bulb	(63)
	**E**: 19%	**E** (MeOH; 1:2; 4°C; 11 min) >> **F** (cheesecloth) >> **E** >> **Ev** (65°C)	NA	**PN**: Shallot **BN**: *A. ascalonicum* **P**: peeled	(30)
	**E**: ~30%	**D** (50°C) >> powdered (80 mesh) >> **E** (apple juice; 1:48; 96°C; 30 min)	Ubon Ratchathani, Thailand	**PN**: shallot **BN**: *A. ascalonicum* L. **P**: bulb	(61)
TEAC	**E**: 0.247 mg TE/g fw	**J** **>>** **F** (sterile gauze) >> **C** (3000 rpm; 4°C; 15 min) >> **F** (Whatman no.1) >> **FD**	Chiangmai, Thailand	**BN**: *A. ascalonicum* cv. *Chiangmai* **P**: peeled bulb	(46)
	**J**: 7834 mg TE/g dw	**J** (Water; minimum amount) >> **F** >> **C** (12000 × g; 20 min; 4°C) >> **FD**	Hsinchu, Taiwan	**PN**: shallot **BN**: *A. ascalonicum* **P**: bulb	(66)
	**E (ethyl acetate)**: 36.25%	**D** (40°C; 3 days) >> **E** (n-hexane ×3 >> ethyl acetate ×3 >> EtOH ×3)	Caringin market, Bandung, Indonesia	**PN**: red onion **BN**: *A. cepa* L. var. *ascalonicum* Backer **P**: bulb	(63)
FRAP	**E**: ~0.04 mM FeSO_4_/g dw	**D** (50°C) >> powdered (80 mesh) >> **E** (apple juice; 1:48; 96°C; 30 min)	Ubon Ratchathani, Thailand	**PN**: shallot **BN**: *A. ascalonicum* L. **P**: bulb	(61)
	**E**: ~0.012 mmol FeSO_4_/g dw	**FD** (5 days) >> **M** (1 mm sieve) >> **E** (Acetone 70%; 1:20; 2 h; 300 rpm) >> **C** (4000 rpm; 15 min) >> **C** (13200 rpm, 5 min)	Tabernal-Zaden, Venhuizen, The Netherlands	**PN**: shallot **BN**: *A. ascalonicum* cv. ‘Creation' F1 **P**: white part	(69)
	**E**: 0.033–0.051 mmol Fe^+2^/g dw	**E** [MeOH; 1:20; modified SOX (Büchi B-811 extraction system)] >> **Ev**	Bejo Zaden, Holland	**PN**: shallot **BN**: *A. cepa* var. *ascalonicum* Backer var. Conservor F1, Bonilla F1 and Matador F1 **P:** bulb	(68)
	**E**: 11.76–19.13 mg GAE/g dw	**E** (chlorine solution; 3:1; 1 min) >> **F** (sieve; 60 min) >> 38% (*w*/*w*) in Keang-hleung paste **PC**: NA	Hatyai, Songkhla province, Thailand	**PN**: shallot **BN**: *A. ascalonicum* L. **P**: bulb	(62)
	**E**: 2.23 mg TE/g extract 0.79 mg GAE/g extract	**J** >> **F** >> **FD** >> **E** (hexane; 1:1; overnight) >> **F** >> **Ev**	local farm, Thailand	**PN**: shallot **BN**: *A. ascalonicum* L. **P**: bulb	(70)
	**E**: 0.27 mg GAE/g sample	**E** (Water; 1:2; 12 h) >> **F** (cheesecloth) >> **C** (4,000 x g for 30 min) >> **Ev** (50°C)	Hat Yai, Songkhla, Thailand	**PN**: shallot **BN**: *A. ascalonicum* L. **P**: bulb	(64)
	**E**: 2.3%	**E** (MeOH; 1:2; 4°C; 11 min) >> **F** (cheesecloth) >> **E** >> **Ev** (65°C)	NA	**PN**: shallot **BN**: *A. ascalonicum* **P**: peeled	(30)
ORAC	**E**: ~40 mg TE/g dw	**FD** (5 days) >> **M** (1 mm sieve) >> **E** (EtOH 70%; 1:20; 2 h; 300 rpm) >> **C** (4500 rpm; 10 min)	Tabernal-Zaden, Venhuizen, The Netherlands	**PN**: shallot **BN**: *A. ascalonicum* (‘Creation' F1) **P**: white part	(69)
	**E**: 34540 mg TE/g dw	**J** (Water; minimum amount) >> **F** >> **C** (12000 × g; 20 min; 4°C) >> **FD**	Hsinchu, Taiwan	**PN**: Shallot **BN**: *A. ascalonicum* **P**: bulb	(66)
	**E**: 3.82 mg TE/g fw	**E** (Water; 1:2)	Mendoza, Argentina	**PN**: shallot **P**: bulb	(60)
TBA reactive substances (TBARS)	**E**: <2 mg MDA within 12 days for 3% extract	**D** (50°C) >> **M** (80 mesh) >> **E** (EtOH 85%; 24 h; r.t.) >> **F** (Whatman No. 1) >> **E** >> **E** >> **Ev** (30° C)	Local herbal store, Gorgan, Iran	**PN:** shallot **BN**: *A. ascalonicum* L. **P**: fruits	(31)
	**UAE**: <0.014 mg MDA/g fish muscle within 5 days of ice storage	**UAE** (Water; 1:10; 15 min at 30°C, 20 kHz) >> **C** (3.000 × *g*; 15 min; 4°C)	BigC supermarket, Nha Trang, Vietnam	**PN**: shallot **BN**: *A. ascalonicum* **P**: bulb	(19)
	**SD**: 0.032 mg MDA/ml oil, 10 μM, 75°C	**SD** (3 h) (y = 2.4 to 3.5 g oil/kg shallot)	Local farms	**PN**: scallion/shallot **BN**: *A. ascalonicum* L./ *Allium fistulosum* auct. **P**: bulb	(18)
	**E**: 0.063 U	**E** (water; 1:1; 3 min; 25°C) >> **F** (Whatman No. 1)	Local market	**PN**: shallot bulb **BN**: *A. ascalonicum* L. **P**: bulb	(71)

*E, extraction; D, drying; SD, steam distillation; F, filtered; FD, freeze-drying; Ev, evaporation; Fract, fractionation; C, centrifugation; J, juice; SLR, solid-liquid ratio (mg/ml); BET, beta carotene equivalent; PE, phenol equivalents; THD, Turbo hydrodistillation; PN, popular name of species; BN, binomial name of species; U, arbitrary units; THD, turbo hydrodistillation; P, part studied*.

### The Influence of Thermally and Non-thermally Processes on Shallot's Antioxidant Activity

Back in 1998, Yin and Cheng compared garlic, bakery garlic, Chinese leek, Chinese chive, onion, scallion, and shallot water extracts in terms of antioxidant capacity. Shallot extract showed potent inhibition of liposome phospholipid oxidation, specifically at a pH range between 4 and 8, from an extract obtained from a heated sample (65°C). The pH treatments at values of 4, 6, and 8 as well as the salt treatments at concentrations of 0.2 or 0.4 M did not affect the antioxidant or prooxidant activity (18). Furthermore, the same research group showed that shallot water extract and shallot oil significantly delayed lipid oxidation in multilamellar phosphatidylcholine liposomes and human red blood cells membranes (71). In a recent study, Lestari et al. (72) documented that the total acidity of shallot increases during the heating period day by day.

Leelarungrayub et al. (70) showed that irradiation of 1:1 water:ethanol solutions of 5 mM of linoleic acid containing a low concentration (24 μg/ml) of shallot extracts resulted in significant inhibition of formation of lipid peroxides, with shallot hexane extract being the most effective. However, the same extracts had no effect on protein oxidation. The antioxidant effect exerted by several plant extracts was evaluated through quantitative atomic spectroscopy and by measuring the levels of malondialdehyde of mice exposed to toxic compounds from air fresheners (73). The research group concluded that shallot extract can overcome the damage produced by air pollutants from air fresheners, although it is not clear from the text to which extent these effects occur.

In a study conducted by Ismail et al. (74), it was found that among all the vegetables (fresh and thermally treated), shallots showed the highest total antioxidant activity followed by spinach, swamp cabbage, cabbage, and kale. Interestingly, shallot's antioxidant activity was not affected by thermal processing (74). The authors ascribe this effect on shallot's high flavonoid content. Indeed, the stability of flavonoids and especially, their glycosides is documented in a study by Chaaban et al. (75). Moreover, it was also observed that despite the total degradation of some flavonoids, the treated solutions still have antioxidant activity.

### The Antioxidant Effect of Polar and Non-polar Extracts From Shallot: *in vitro* Studies

In the study of Leelarungrayub et al. (70), hexane extract from shallot exerted the highest antioxidant activity when compared to water extract, bulb pressings, and commercial products. This effect can be ascribed to the higher quantity of diallyl disulfides and non-polar compounds extracted in this media. Also, Chaithradhyuthi et al. (30) showed that methanol extract of shallot exerted the highest radical scavenging activity, having the highest composition in phenolics (0.98 mg PE/g) when compared to garlic and onion. The same trend was observed by Fidrianny et al. (63), aqueous shallot extract yielding the highest content in phenolics and flavonoids when compared to onion and garlic. On the other hand, aqueous shallot extract exerted a moderate peroxynitrite-scavenging ability (40.2%), probably due to the low content of bioactive compounds extracted in the extraction media (66). Bernaert et al. reported a moderate antioxidant activity as well. The antioxidant capacity of *A. ascalonicum* bulbs was evaluated through ORAC and DPPH [extracts were obtained by 70% aqueous ethanol solution (*v*/*v*)], and FRAP [extract was obtained by acetone-water (70:30) with 0.1% formic acid] assays. The main limitation of the study was the fact that only bulbs of shallot were assessed, whereas, for the other seven *Allium* species the green leaves were evaluated as well. The study revealed that, in general, leaves have significantly stronger antioxidant properties (69). Beretta et al. (60) documented that among 6 *Allium* species, the aqueous shallot extract exerted the 2nd best antioxidant activity, after chives. They further documented that phenolics, as a whole, are largely responsible for antioxidant activity, with broad variation observed among the contributions of individual phenolic compounds. Another group of researchers tested the antioxidant effects of the powder of shallot juice extract on human vascular endothelial cell lines (EA.hy 926) subjected to oxidative stress (Figure 2j). The extract reduced the iron-induced malondialdehyde production in a dose-dependent manner and also significantly reduced the H_2_O_2_-induced ROS production at a low concentration (<200 μg/ml) (46).

### The Antioxidant Effect of Shallot Essential Oil

The essential oil of shallot showed an important antioxidant activity at 75°C. The addition of shallot oil significantly reduced the MDA formation in isolated human RBC membranes (18). Assessing the antioxidant activity through the DPPH test, Mnayer et al. (21) obtained an IC_50_ of 2.70 mg/ml. It seems that the methods employed to extract the volatile compounds from shallot lead to their degradation, consequently, the antioxidant activity is significantly lowered.

### The Influence of Storage Conditions on Antioxidant Activity of Shallot

Interestingly, Pudzianowska et al. showed that there was no significant influence of storage conditions on antioxidant activity of shallot assessed with DPPH test, while antioxidant activity assessed with FRAP test was significantly influenced. The bulbs stored in an atmosphere of 5% CO_2_ + 2% O_2_ were characterized by the highest FRAP values (68). Aiming to evaluate the effect of gamma irradiation on antioxidant activity exerted by shallot, Sinuraya et al. (24) found that neither control nor irradiated shallot genotypes exert a significant antioxidant capacity.

### The Influence of Extraction Method on Antioxidant Activity of Shallot

Regarding the influence of extraction method and solvent type on antioxidant activity, Saptarini et al. aimed to identify the best extraction method (maceration, percolation, reflux, and Soxhlet method) and the most suitable solvent (water, ethyl acetate) in terms of antioxidant activity exerted by shallot extracts using DPPH test. They concluded that the highest antioxidant activity could be achieved through the reflux method using ethyl acetate as solvent (67). This result can be ascribed to a higher extraction yield of phytochemicals with potent antioxidant activity (e.g., sufloxides) generated by a longer contact of plant matrix with the solvent. Additionally, the group of Crnivec et al. (15) aimed to examine various parts of the plants (edible/inedible waste/outer skin of onion), as well as extraction in water/ethanol and by shaking/sonication. They concluded that ethanol extracts of the waste fraction had the highest quercetin content and antioxidant capacity.

### The Antioxidant Potential of Shallot for Food Preservation Applications

Shallot has a great potential for food industry application due to its antioxidant capacity. Raeisi et al. showed that shallot extract could extend the shelf-life of semi-fried coated rainbow trout (*Oncorhynchus mykiss*) filets. Researchers measured the lipid oxidation indicators during a 15 day timeframe, concluding that shallot extract contributed to the significantly higher sensory quality and overall acceptability and that shallot could be applied as a natural preservative for extending the shelf-life of fish products (31). These results are further supported by a recently published article which investigated the antioxidant activity of hydrophilic extracts of shallot on round scad (*Decapterus punctatus*) during ice storage. It was found that shallot extract exhibited good antioxidant activity concerning DPPH radical scavenging, total reducing power, and lipid peroxidation inhibition. Round scad treated with either shallot or garlic extracts kept their natural sensory characteristics accepted for food-grade longer than 4 days of ice storage, compared with the control (without treatment) (Figure 2k) (19). A recent study reported the development of active packaging film using sodium alginate and carboxymethyl cellulose along with shallot waste extract. The study highlights that the film developed with shallot stalk extract can be used for the wrapping of fresh-cut fruits and vegetables. It also prevents browning and maintains the overall quality of control and shallot peel incorporated film (76). The potential of shallot waste to be used in the packaging industry is confirmed by another recent study, which developed an innovative method of xyloglucan extraction from shallot stalk. The film obtained from xyloglucan can be used as an alternative to plastic films (77).

Another interesting research showed that temperature treatment and shallot supplementation can improve the quality of apple juice by increasing its antioxidant capacity in terms of DPPH radical scavenging activity and FRAP (61).

### Antioxidant Activity of Isolated Compounds From Shallot

**Figure 3:**
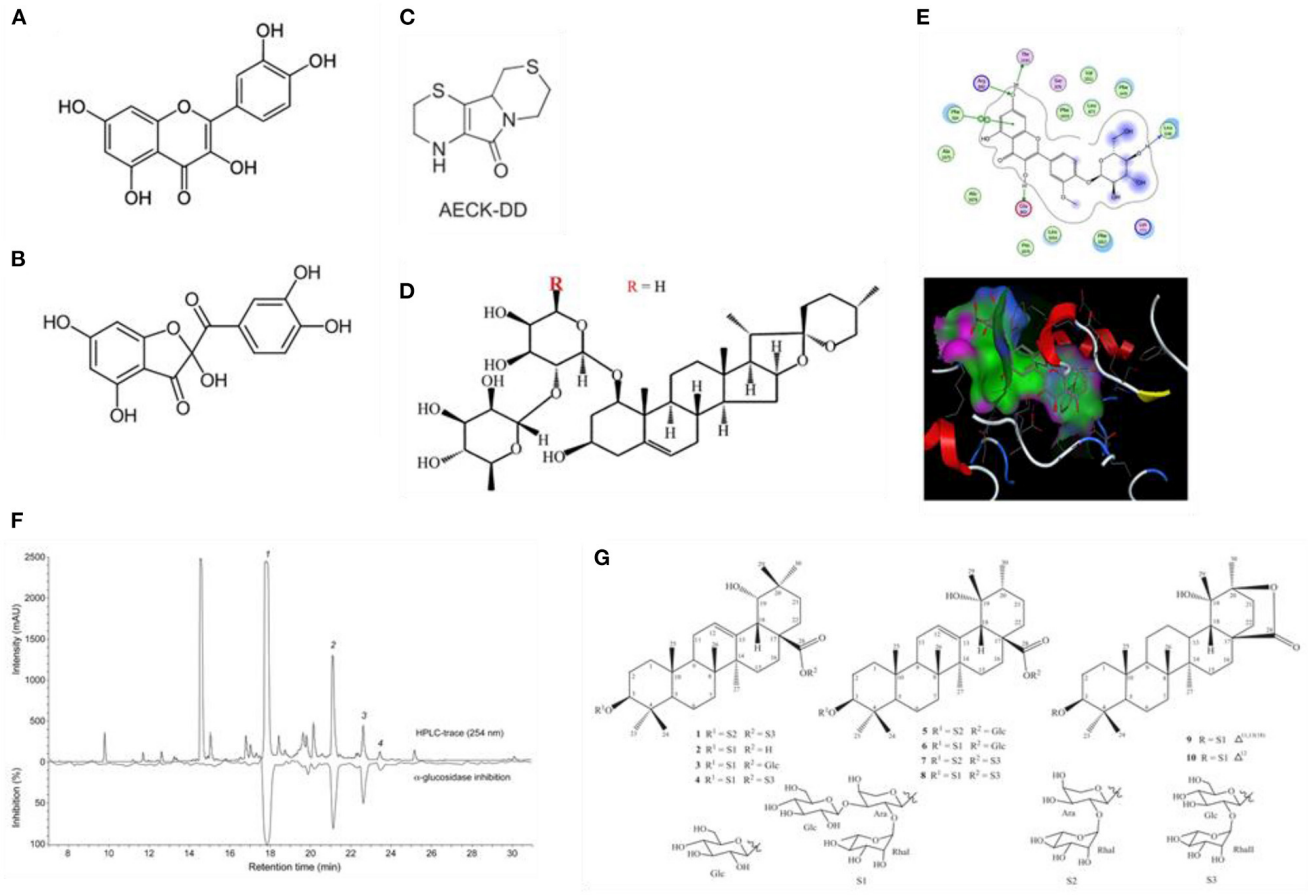
Chemical structure of **(A)** quercetin (3,5,7,3′,4′-pentahydroxyflavone); **(B)** the quercetin-derived benzofuranone [2-(3,4-dihydroxybenzoyl)-2,4,6-tri-hydroxy-3(2*H*)-benzofuranone]. **(C)** Aminoethyl cysteine ketimine decarboxylated dimer (AECK–DD); **(D)** Cepa2; **(E)** binding mode of isorhamnetin-3-glucoside with xanthine oxidase. **(F)** High-resolution α-glucosidase inhibition profile of the ethyl acetate extract of the peel of *A. ascalonicum* with overlaid HPLC chromatogram at 254 nm. Peaks numbered **1**–**4** (corresponding to compounds **1**–**4**) correlated with α-glucosidase inhibition and were analyzed by HPLC-HRMS-SPE-NMR. **(G)** Chemical structures of compounds 1–10 isolated from shallot screened for anoctamin inhibitory capacity. Compounds **2** and **9** exerted the highest anoctamin inhibitory potential (78–83).

Recently, Fuentes et al. documented the occurrence of the quercetin oxidation metabolite 2-(3,4-dihydroxybenzoyl)-2,4,6-trihydroxy-3(2H)-benzofuranone (Figures 3A,B). Among 20 quercetin-rich plant foods, it was only detected in the dry outer scales of onions and shallots. The finding was followed by antioxidant evaluation and cytoprotective properties of shallot aqueous extracts. Results showed that shallot extract has protected ROS-exposed Caco2 cells against oxidative (78%) and cellular (90%) damage at a 3 μg/L concentration (78).

Interestingly, Tsikas et al. found that among many other plant species studied, the shallot is the only one that contains aminoethylcysteine ketimine decarboxylated dimer (AECK–DD, Figure 3C), a metabolite of cysteamine that has been reported to be present in human plasma and urine, in the mammalian brain and many common edible vegetables possessing potent antioxidative properties. The concentration in which the compound was found in shallot is ~6.8 pmol/g fresh tissue (79).

### The Inhibition of Lipid Oxidation Exerted by Shallot Extract

The aqueous shallot extract can inhibit LDL oxidation, thus reducing the incidence of atherosclerosis (84). The same research group found a similar effect for hydroalcoholic shallot extract at concentrations ranging from 4 to 12 mg/ml (85).

### Anti-inflammatory Activity

Tuntipopipat et al. aimed to assess the anti-inflammatory potential of 13 common Thai spices. They revealed that shallot extract does not have anti-inflammatory activity on LPS-Activated RAW 264.7 cells (86). Similarly, the inhibition of NO synthesis in the LPS-induced macrophages cells produced by shallot extracts was very weak (IC_50_ = 633.510 μg/ml) compared to other ingredients from a traditional Thai paste (Keang-hleung) (64). Contrary, Ounjaijean et al. (46) showed that the shallot juice extract exerted a good anti-inflammatory effect on LPS-induced endothelial cell lines.

Moreover, on an acetic acid-induced vascular permeability model, the shallot extract showed an excellent anti-inflammatory effect, comparable to the standard currently used, indomethacin (87). Furthermore, Kantangkul et al. (62) reported a strong anti-inflammatory effect of a popular Thai dish that incorporates shallot as well by the inhibition of NO production, although the observed effect is not solely attributable to shallot itself, but rather to the complex mixture of different ingredients. Later, Chakree et al. (64) evaluated the antioxidant capacity of the ingredients from Keang-hleung paste extract, emphasizing that shallot is contributing to a lesser extent to the overall antioxidant capacity exerted by this traditional dish prepared in Thailand.

#### Inflammatory Arthritis

Gout is a form of inflammatory arthritis characterized by elevated levels of uric acid in the blood, leading to intense joint pain, the formation of tophi, kidney, and liver damage. Allopurinol, a xanthine oxidase inhibitor, is the standard drug used to lower uric acid levels. Two papers were found to assess the beneficial effects of shallot in gout treatment/prevention. Aiming to optimize the extraction process of shallot, a recent study revealed that the shallot ethanolic (50%) extract from skin processed at 85°C for 120 min can inhibit xanthine oxidase (IC_50_ = 21.09 μg/ml). To explain the role of flavonoids on xanthine oxidase inhibitory activity of shallot, molecular docking between 9 flavonoids which was isolated from shallot and xanthine oxidase was performed. The study revealed that among them, isorhamnetin-3-glucoside was the most potent compound with the lowest binding energy (Figure 3E) (80).

### Remarks and Future Outlooks

Taken together, these results support the idea of using shallot preparations in NCD prevention and as an adjuvant treatment. However, several limitations can be identified among the vast majority of these studies: the results of *in vitro* assays have been erroneously extrapolated to the therapeutic application of plant antioxidants without undertaking sufficient *in vivo* studies. Moreover, the results are expressed using various measurements units (e.g., for DPPH assay, the results are expressed either using IC_50_ value, mg Trolox Equivalents/g dw or fw, mg Gallic Acid Equivalents/g dw or fw, or expressed as a percent of inhibition), making a comparison between the extraction and processing methods almost impossible to perform. To date, no study assessed the antioxidant status change exerted by shallot in a clinical trial, whereas for garlic there are at least 9 clinical studies assessing this potential (88). An interesting observation is that the antioxidant potential of shallot is not influenced by thermal processing neither in a positive manner nor in a negative one, taking into account that cooking influences the antioxidant activity of plants in some way (89). The research community should conduct more studies to confirm or infirm this and other observations by performing more *in vitro* studies followed by *in vivo* ones and, if the results are promising, by clinical trials.

## Cardiovascular Diseases Management and Prevention

In 2019, 7 out of 10 causes of death were associated with a non-communicable chronic disease (NCD), with 44% of all deaths worldwide (58). Cardiovascular diseases (CVDs) are the number 1 cause of death globally: more people die annually from CVDs than from any other pathology. One of the most important causes of these heterogeneous diseases is an elevated level of cholesterol which most often leads to the development of atherosclerosis (90). In the following part, we aim to summarize documented effects exerted by shallot on hematological and lipid profile, antiplatelet activity, and data regarding ischemic stroke prevention. For more details regarding the experiments, Table 3 should be consulted.

**Table 3:** Summary of main cardiovascular, antidiabetic, cytotoxic and other pharmacological effects exerted by shallot extracts.

**Pharmacological effect**	**Model**	**Results**	**Extract**	**Zone**	**Species**	**References**
**Cardiovascular effects**						
Effect on hematological profile	*In vivo* **A/M**: NA **O**: male albino rats **PC**: NA	(**E**) 0.05, 0.1 and 0.2 g/kg ↓ Total cholesterol, HDL, LDL ↓Red blood cells parameters ↑ Platelet count, percent neutrophil ↕ triglycerides	**E** (EtOH)	Ilorin, Nigeria	**BN:** *A. ascalonicum* **P**: dried leaves	(91)
	*In vivo* **A**/**M**: streptozotocin-induced diabetes **O**: 40 wistar albino (*Rattus norvegicus*) rats **PC**: acarbose	(**Snack**) 14 days ↓ Packed cell volume (PCV), hemoglobin concentration (Hbc), white blood cells (WBC), red blood cells (RBC), lymphocytes levels	**D (**40°C; 96 h) >> **M** (60 mm mesh sieve) >> **Snack** (25%)	Shasha market, Akure, Ondo State, Nigeria	**PN**: Shallot **BN**: *A. ascalonicum* **P**: bulb	(65)
Effect on lipid profile	*In vivo* **A/M**: Atherogenic feed → hypercholesterolemia **O**: *Rattus novergicus* **PC**: Atorvastatin	(**F**): 0.2 g/kg ↓ Total cholesterol with 20.44% (7th day); 37% (14th day) and 49.58% (21st day) (PC) 20 mg ↓ Total cholesterol with 15.62% (7^th^ day); 25.84% (14^th^ day) and 36.47% (21^st^ day)	**F** (15 days, 50–80°C)	NA	**PN:** red onion **BN:** *A. cepa* L. var *aggregatum* **P**: fresh, without peeling	(92)
Antiplatelet activity	*In vitro* **A/M**: electrical impedance aggregometry **O**: Blood collected from 2 healthy donors **PC**: NA	(**E**) IC_50_ = 18.9 mg fw/ml whole blood	**E** (Water; 1:2)	Mendoza, Argentina	**PN**: Shallot **P**: peeled bulbs	(60).
	*In silico* **A/M***:* molecular docking	**UAE** (compounds identified): P2Y12 inhibitory activity	**UAE** (EtOH 96%; 1:4.375; 40°C, 30 min) >> **F** >> **Ev**	traditional markets	**PN**: Shallot **P**: skin	(93)
Ischemic stroke prevention	Cross-sectional observational 125 patients	No good correlation between shallot intake and brachial artery flow-mediated dilatation	Food frequency questionnaire	China	**PN**: Shallot **P**: NA	(94)
**Antidiabetic effects**						
α-glucosidase inhibition	*In vitro* **A/M**: High-resolution α-glucosidase profiling **PC**: NA	(**UAE**) IC_50_ = 0.012 mg/ml	**D** (sun-dried or oven-dried at 40°C) >> **UAE** (EtOAc; 1:10.86; 3 min shaking; 2 h sonication; r.t.)	Denmark	**PN**: Shallot **BN***: A. ascalonicum* **P**: peel	(81)
Effect on glucose levels	*In vivo* **A/M**: alloxan-induced diabetes Wistar rats **PC1**: acarbose (20 mg/kg) **PC2**: glibenclamide (5 mg/kg) **PC3**: metformin (100 mg/kg)	(**E**) 0.5 g/kg ↓PBG 5.7% (~**C2**) – short term (3 h) ↓PBG 32% (~**C3**) – long term (3 weeks) ↑regulation of Glut-4 and Insulin genes	**E** (MeOH 80%; 1:1.5; Soxhlet; 72 h) >> **F** >> **Ev** (y = 17%)	Alborz mountain in the north of Tehran, Iran	**PN**: Persian shallot **BN***: A. ascalonicum* L. **P**: bulb	(95)
Effect on antioxidant enzyme status Effect on lipid profile	*In vivo* **A/M**: alloxan-induced diabetes **O**: Wistar rats **PC**: metformin (100 mg/kg)	(**E**) ↑ SOD (65%), ↑ GPX (43%) ↑ CAT (55%) ↓VLDL (24%)	**D** >> E (MeOH 80%; 1:1.5; Soxhlet; 72 h) >> **F** >> **Ev** (y = 17%)	Tehran province of Iran	**PN**: Persian shallot **BN**: *A. ascalonicum* L. **P**: bulb	(96)
Effect on histology of liver	*In vivo* **A/M**: alloxan-induced diabetes **O**: Wistar rats **PC**: metformin (45 mg/kg BW)	(**E**) 0.25 g/kg ↑histopathological feature of liver	**NA**	NA	**PN**: Onion/shallot **BN**: *A. ascalonicum* L **P**: NA	(97)
Effect on glucose levels	*In vivo* **A/M**: Fructose-induced insulin resistance **O**: 34 male albino Wistar rats **PC**: NA	(**E**) 0.75 g/kg (4th week) ↕Fasting insulin resistance index; ↕Intraperitoneal glucose tolerance test (8th week) ↑Intraperitoneal glucose tolerance test; ↓Fasting insulin resistance index	**E** (0.9% saline; 1:0.66; 15 min) >> **F** (3 times) >> + 0.9% saline (1:1.33)	Local market in Mashhad, Iran	**PN**: Shallot **BN:** *A. ascalonicum* L **P**: bulb	(98)
Effect on glucose levels	*In vivo* **A**/**M**: streptozotocin-induced diabetes **O**: 40 wistar albino (*Rattus norvegicus*) rats **PC**: acarbose	(**E**) 30% to 59% reduction in blood glucose	**D (**40°C; 96 h) >> **M** (60 mm mesh sieve) >> **Snack** (25%)	Shasha market, Akure, Ondo State, Nigeria	**PN**: Shallot **BN**: *A. ascalonicum* **P**: bulb	(65)
Effect on lipid profile	Parallel randomized clinical trial **O**: 48 participants **PC**: NA	(**Fresh**) 2 g shallot/100 g yogurt ↓ TG, TC and LDL-C ↕ FBS	Fresh shallot	Caleh Company. Caleh, Isfahan, Iran	**PN**: Shallot **P**: NA	(99)
**Cancer-related effects**						
Cytotoxicity	*In vivo* **A/M**: Antitumor activity **O**: BDF1 mice **PC**: NA	(**E**) 0.266–0.5 g/kg P388 leukemia ↑ % of median survival time	**E** (Water; 1:5; 5 min) >> C (1 h, 2000x g) >> **FD**	Local market, USA	**PN**: Shallot **BN:** *A. ascalonicum* **P**: bulb	(100)
	*In vitro* **A/M**: MTT assay HepG2 **PC**: NA	(**E**) IC_50_ = 0.050 mg/ml	**E** (EtOH; 1:1; one night) >> F (no 1) >> **C** (16000 rpm; 4°C) >> **D** (80°C – 20 min; 50°C – 30 min)	Local vegetable markets, Thanjavur, Tamil Nadu, India	**PN**: Shallot **BN**: *A. ascalonicum* **P**: bulbs	(101)
	*In vitro* **A/M**: Alamar blue assay; flow cytometry; Western blot and Caspase 3 activity kit **PC**: NA	↓ Bcl-2 and surviving ↑ Bax, Bad, Apaf-1, p53 and Caspase 9	NA	NA	**BN:** *A. ascalonicum* **P**: NA	(102)
	*In vitro* **A/M**: Trypan blue exclusion assay and activity of lactate dehydrogenase method **O**: K562, Jurkat, Wehi164, and HUVEC **PC**: NA	(**E**) K562 (IC_50_ = 100 μg/ml) Jurkat (IC_50_ = 100 μg/ml) Wehi164 (IC_50_ = 400 μg/ml) HUVEC (IC_50_ = 1600 μg/ml)	**E** (Water; 1:1; one night) >> **F** >> **C** (16,000x g; 4°C; 30 min) >> **FD** (y = 27.4%)	local vegetable markets at Kermanshah, Iran	**PN**: Shallot **BN***: A. ascalonicum* **P**: bulbs	(87)
	*In vitro* **A/M**: MTT **O**: P3U1 cell line **PC**: NA	(**Cepa2**) 0.050 mg/ml 91.13% reduction in P3U1 cell viability 12 h apoptosis of the Cepa2-treated P3U1 cells in a time course-dependent manner	Complex–compound isolation (Cepa2/alliospiroside A)	NA	**PN**: Shallot **BN**: *A. cepa* L. Aggregatum group **P**: dry roots	(82)
Desmutagenicity	*In vitro* **A/M**: Mutagenicity inactivation assay **PC**: NA	(**J**) ↓ mutagenic activity effect by tryptophan-pyrolysates	**J** **>>** **C** (9000x g; 30 min)	Misima, Japan	**PN**: Shallot **P**: NA	(103)
Angiogenesis	*In vitro* **A/M**: human umbilical vein endothelial cells (HUVECs) **PC**: quercetin *In vivo* **A/M**: chicken chorioallantoic membrane (CAM) model	(**E4**) IC_50_ = 1 μg/ml (HUVEC) (**PC**) IC_50_ = 10 μg/ml (**E4**) 3 ng/egg (see Figure XXB) 10 ng/egg (see Figure XXC)	**E1** (EtOH 50%; 24 h) >> **C** (12,000x g;20 min; 4°C) >> **Ev** **>>** **E2** (Water) >> **E3** (n-hexane) >> **E4** (ethyl acetate) >> **E5** (n-butanol) >> **Ev**	local vegetable market at Kermanshah (Iran)	**PN:** Shallot **BN**: *A. ascalonicum* **P**: NA	(47)
	*Ex vivo* **A/M**: aorta ring model **O**: Wistar male rats **A/M**: Cytotoxicity: trypan blue assay **PC**: NA	(**E**) 50–800 μg/ml ↑ anti-angiogenetic effect	**E** (Water)	NA	**PN**: Shallot **BN**: *A. ascalonicum* **P**: NA	(104)
Clinical studies	Case control **O**: 627 patients	Inverse association between shallot intake and gallbladder cancer (OR: 0.81, 95% CI: 0.68–0.97)	**NA**	Shanghai, China	**PN**: Shallot	(105)
	Case control **O**: 220 patients	Intake of shallot and garlic associated with a reduced risk of multiple myeloma (OR = 0.60, 95% CI: 0.43–0.85)	**J**	Northwest China	**PN**: Shallot	(106)
	Case-case control *Ex vivo* **A/M**: S-P immunohistochemical technique (expression of CD44v6)	Expression of CD44v6 is correlated with gastric cancer ≥7 times/week consumption of shallot and garlic →	**J**	China	**PN**: Shallot	(107)
	Retrospective questionnaire	Eating shallots → protective effect against breast cancer	**J**	Maoshan Municipal People' s Hospital, China	**PN**: Shallot	(108)
**Other pharmacological effects**						
Xanthine oxidase inhibitory activity	*In vitro* **A/M**: Xanthine oxidase assay **PC**: Quercetin	(**E**): IC_50_ = 3.23 μg/ml (**PC**): IC_50_ = 2.69 μg/ml	**E** (EtOH 50%;1:125; 85°C; 30 min) >> **Ev** (50°C; 80 mbar)	Kinh Mon, Hai Duong, Vietnam	**BN***: A. cepa* var. *aggregatum* G.Don **P**: skin	(80)
Anti-tyrosinase Anti-melanogenic	*In vitro* (mushroom) *In vivo* (B16F10 cell line) **PC**: Kojic acid	(**E**) IC_50_ = 22.79 ± 3.49 mg/ml (mushroom) IC_50_ = 12.40 ± 1.08 mg/ml (B16F10) ↓ melanin (B16F10) (**PC**) %I = 41.85% (20 μM)	**D** >> **E** (EtOH 20%; 40°C; 4 h) >> **Ev**	Phayao, Thailand	**BN***: A. ascalonicum* **P**: NA	(109)
Anoctamin-1 inhibitory activity	*In vitro* **A/M**: yellow fluorescent protein reduction assay **PC**: Ani9	(**E**) 30 μM Alliumascaside B: 28.9 ± 0.85% Kudinoside D: 26.2 ± 0.65% (**PC**) 3 μM: 97.5% (%I)	Complex–compound isolation	Hanoi City, China	**BN***: A. ascalonicum L*. **P**: rhizomes	(83)
Uric acid level lowering	*In vivo* **A/M**: potassium oxonate-induced hyperuricemia **O**: male Sprague-Dawley rats **PC**: Allopurinol	(**E**) 10.5 g/kg/day (PC) 0.005 g/kg/day ↓ Uric acid levels ↓ Histological changes in liver ↕ Histological changes in kidney	**J** (Water; SLR 1:1)	Grand Union Supermarket, Serdang, Malaysia	**PN**: red onion **BN**: *A. cepa* var. *aggregatum* G. Don **P**: edible part	(110)
Nephrotoxicity protection	*In vivo* **A/M**: Cyclosporine-induced nephrotoxicity **O**: Male Wistar rats **PC**: 1,1,3,3-tetramethoxypropane (MDA, GSH)	(**E**) 1 g/kg/day ↑Renal function ↓Oxidative stress markers (MDA, GSH) ↓Morphological changes	**J** >> **F** >> **FD**	Local farm, Thailand	**PN**: Shallot **BN**: *A. ascalonicum* L. **P**: bulb	(111)
Hepatoprotection	*In vivo* **A/M**: (**a**) Acute toxicity test (**b**) Ethanol-induced Liver Injury **O**: male ICR (Imprinting Control Region) mice **PC**: silymarin	(**a**) (**E**) 2 g/kg/day - no toxicity (**b**) 0.2 g/kg/day (**E**) and 10 mg/kg/day (PC): ↓AST, ALT, GGT, ALP	**E** (Water; SLR 1:10; 5 min; 72 h) >> **F** (gauze) >> **FD**	Local market, Suphanburi Province, Thailand	**PN**: Shallot **BN**: *A. ascalonicum* **P**: peeled bulbs	(46)
Spermatogenesis	*In vivo* **O**: Balb/C Mice **PC**: NA	(**E**) 0.8 mg/kg/day ↑ Spermatogonia ↑ Primary spermatocytes ↑ Spermatids ↑ Internal and external diameters of tubules and germinal layer area	**E** (EtOH; SLR 1:0.714; 4 days) >> **F** >> **Ev**	Chaharmahal and Bakhtiari province, Iran	**BN***: A. cepa* var. *ascalonicum* **P**: NA	(112)
	*In vivo* **A/M**: streptozotocin-induced diabetic mice **O**: Adult male mice (ICR strain) **PC**: glibenclamide	(**J**) 1,000 mg/100 g BW (PC) 1 mg/100 g BW ↑ gonadal index;↑ sperm concentration;↑ number of viable sperms; ↑ number of motility sperms	**J**	Khon Kaen province, Thailand	**BN***: A. ascalonocum* **P**: fresh aged bulbs	(113)
Wound Healing	*In vivo* **A/M**: Excision wound model **O**: Sprague Dawley rats **PC**: Terramycin	(**E**) 10%; 20% (*w*/*w*) and (PC) ↑ Wound healing Fully recovered epidermis in 8 days, compared to control (12 days)	**E** (95% EtOH; SLR = 1:1; 1 night, r.t.) >> **F** >> **Ev** >> **FD**	Thai market in Pathumthani, Thailand	**PN**: Shallot **BN***: A. ascalonicum* Linn. **P**: dry bulbs	(20)
Mucolytic	*In vitro* **A/M**: Mucolitic activity test duck egg albumen **PC**: N-Acetylcysteine	(**E**) 25% and (PC) 0.2% ↓Viscosity of albumen	**E** (EtOH 96%; 1:5; 1 h, 40°C) >> **F** >> **Ev** (40 ° C)	“Market”, Indonesia	**PN**: shallot **BN***: A. ascalonicum* L. **P**: peeled bulbs	(114)
Anti-cough	*Clinical trial* **A/M:** acute cough **O**: 14 patients	(**Mixture**) 3.23%	**Mixture**	Thai herbal shop	**PN**: Shallot **BN**: *A. ascalonicum L*.	(115)
Iron availability	*In vitro* **A/M**: iron availability assay by (116); **PC**: NA	(**E**) ↓ Iron dialyzability by 50–80%	**J** **>>** **FD**	Ubon Ratchathani province, Thailand	**PN**: shallot **BN***: A. ascalonicum* **P:** bulb	(117)
	*In vitro* **A/M**: iron availability assay after enzymatic treatment **PC**: NA	(**E**) ↑ Iron availability (18.9%—raw rice; 23.3—cooked rice; 48.2%—raw grains)	**J**	Local market, China	**PN**: shallot **BN**: *A. ascalonicum* **P**: bulb	(118)

*A/M, assay/model; O, organism; PC, positive control; E, extract; D, drying; J, juice; C, centrifugation; Ev, evaporation; P, plant part; F, filtration; FD, freeze-drying; NA, not available; UAE, ultrasound-assisted extraction*.

### *In vivo* Studies on the Cardiovascular Effect of Shallot

A study by Owoyele et al. (91) reported that shallot extract administrated for 21 days in white albino rats decreased total cholesterol (TC), high-density lipoprotein cholesterol (HDL), and low-density lipoprotein cholesterol (LDL) levels, but had no significant effect on triglyceride levels. However, the research team hasn't used a positive control, a substance with well-known effects on lipid profile, thus bringing limitations to the reliability of observed effects of the intervention. Another similar study that used atorvastatin as positive control documented that a dose of 200 mg/kg of fermented shallot has a strong effect in decreasing cholesterol levels (92), but the methodology used for extracting the essential oil is lacking and besides that, the authors did not evaluate the influence of crude extracts on total cholesterol level. Moreover, the time required for the fermentation process is very long and there is no scientific support mentioned in choosing this process.

### *In vitro* Studies on the Cardiovascular Effect of Shallot

A study aiming to evaluate the *in vitro* antiplatelet activity of several *Allium* species revealed that garlic and shallot were the strongest agents with antiplatelet activity. Besides, significantly positive correlations were found between the *in vitro* antiplatelet activity and total organosulfur and phenolic (TP) content (60). However, the study fails to provide reproducible data, as the scientific name of the shallot is not provided, and the extraction procedure is very scarce in detail. The results are, however, confirmed by another recent study which reports that the methanolic extract contains several compounds that in molecular docking studies showed a good inhibitory capacity on the P2Y12 receptor. Moreover, the ADMET prediction data displayed that the compounds in the extract have good absorption so that they can be used in the oral and transdermal routes (93).

### Clinical Studies on the Cardiovascular Effect of Shallot

In contrast, a study aiming to evaluate the effects of several *Allium* species on endothelial function of 125 patients with prior ischemic stroke due to athero-thrombotic disease revealed that there is a weak correlation between daily shallot intake and brachial artery flow-mediated dilatation, a parameter that reveals the stage of the disease. Interestingly, a better correlation was found between daily *Allium* and garlic intake and the brachial artery flow-mediated dilatation amelioration (94). The main limitations of this study are the small sample size, the scientific names of the species are not provided and it is not known whether the *Allium* species were consumed raw or cooked.

## Diabetes Treatment and Prevention

According to the World Health Organization (WHO), about 422 million people worldwide have diabetes, most of them living in low-and middle-income countries, and 1.6 million deaths are directly attributed to diabetes each year (119). Thus, it became very important to find new sources of antidiabetic agents, inspired by the bioactive compounds in plants. In this regard, several studies were conducted to assess the antidiabetic potential of *A. ascalonicum* extracts.

### Inhibition of α-Glucosidase

A target enzyme in diabetes is α-glucosidase, located in the brush border of the small intestine. Its inhibition lowers the rate of glucose absorption through delayed carbohydrate digestion and extended digestion time. Kongstad et al. investigated the phytochemical profile of shallot using a combined technique of high-resolution α-glucosidase inhibition profiling and HPLC-HRMS-SPE-NMRS for the investigation of antidiabetic principles in crude shallot extracts. They found that the quercetin (1), its dimer (2), trimer (3), and tetramer (4) are the main compounds that act as α-glucosidase inhibitors in *A. ascalonicum* peel extracted in ethyl acetate, the inhibitory activity decreasing in this order (1 > 2 > 3 > 4) (Figure 3F) (81). However, as the authors mention in the “Discussion” part, it is not clear from the data available in the manuscript whether the identification of quercetin trimer and tetramer is accurate.

### Antidiabetic Potential of Shallot Extracts: *in vivo* Studies

Several *in vivo* studies were conducted to assess the effect of *A. ascalonicum* extracts on diabetic animal models. Sani et al. (96) documented that the methanolic extract of shallot increases the activities of SOD (60%), GPX (43%), and CAT (55%), three ROS-scavenging enzymes whose levels are altered in diabetes because of the hyperglycemia. The same study reported that the lipidic profile of alloxan-diabetic rats was improved, namely, the VLDL levels decreased by 24% compared to control. Additionally, the effect of methanolic extract of *A. ascalonicum* on alloxan-diabetic rats was investigated by the same research group in comparison to the effects of antidiabetic drugs such as acarbose, glibenclamide, and metformin. The extracts showed a significant reduction of PBG (postprandial blood glucose) similar to glibenclamide in a short timeframe and similar to metformin after 3 weeks of treatment. Moreover, the study shows that in addition to the inhibition of α-glucosidase, the extract up-regulates the expression of *Ins* and *Glut-4* genes (95). However, an important limitation of this study is that the effect of standard drugs on gene expression was not evaluated. A more recent study analyzed the ability of shallot-enriched amaranth-based extruded snacks to modulate carbohydrate hydrolyzing enzymes in streptozotocin-induced diabetic rats. Animals fed with snacks showed ameliorative effects on hematological parameters, the attenuated elevation of enzyme activities in kidney and liver homogenates, and displayed decreased α-glucosidase/α-amylase activities (65).

One of the main mechanisms involved in diabetes physiopathology is cells' resistance to insulin. The study conducted by Jalal et al. (98) showed that the fasting blood glucose in fructose-induced insulin resistance animals decreased significantly. Interestingly, the study was carried out in comparison to garlic extract and the results showed that the shallot extracts have the ability to improve the intraperitoneal glucose tolerance and diminish the fasting insulin resistance index, whereas the garlic extract did not have a significant effect on these parameters.

Recent studies documented a strong association between male infertility and diabetes (120). Luangpirom et al. (113) documented that the treatment with shallot juice of streptozotocin-induced diabetic mice attenuated the impaired testicular function. Specifically, it was observed an increase in gonadal index and sperm quality. These results are confirmed by another study that evaluated in an *in vivo* experiment the effect of shallot hydroalcoholic extract on sperm production. The results pointed out a significantly increased spermatogonia, primary spermatocytes, spermatids, and internal and external diameters of tubules and germinal layer area in experimental groups treated with shallot extract (112).

A study aiming to evaluate the effects of yogurt and yogurt plus shallot intake on lipid profile in type 2 diabetic women showed that triglyceride and total cholesterol concentrations decreased more in participants who consumed yogurt plus shallot than those who consumed yogurt. Moreover, LDL-C level of participants who were in yogurt plus shallot group was lower than that of participants in yogurt group, but this difference was marginally significant (99). The limitation of this study is that the authors did not mention the scientific name of the shallot species considered. Also, the process of obtaining the yogurt is not well described in the manuscript, thus making the results irreproducible.

All these results suggest that through various mechanisms, the shallot extract has beneficial effects on diabetes prevention or can be used as an adjuvant treatment for diabetes (Table 3).

## Cancer Prevention and Cytotoxic Activity

*Allium* species are wellknown for their composition in compounds that have an important cytotoxicity effect. Some of the most important findings regarding the anti-cancer activity of *Allium* species' extracts can be found in the comprehensive review compiled by Asemani et al. (121). Moreover, Bommareddy et al. (122) specifically discuss the role of organosulfur compounds derived from *Allium* vegetables in cancer prevention and therapy. However, in this part, we will focus on the main findings reported on the cytotoxic activity of *A. ascalonicum* extracts.

### Cytotoxicity Effects

Back in 1973, it was first postulated that the aqueous extracts from shallot have significant activity against P388 leukemia in mice (100). Since then, many other studies assessed the cytotoxic ability of shallot preparations (see Table 3). For example, it was reported that the shallot extracts from both dry and fresh bulbs have a cytotoxic effect on liver cancer cell line HepG2 with an IC_50_ of 50 mg/ml (101). However, in this study, there was no positive control used to compare the results. Another study aiming to elucidate the mechanisms involved in the cytotoxic activity of *A. ascalonicum* extracts on cell line HepG2 showed that the expression of Bcl-2 and survivin was downregulated, whereas the expression of Bax, Bad, Apaf-1, p53, and Caspase 9 was upregulated, meaning that the apoptosis is induced by this extract through the mitochondria-mediated pathway (102).

The aqueous extract of *A. ascalonicum* bulbs exerted a high cytotoxic activity on cell lines Jurkat and K562 (IC_50_ of 0.1 mg/ml after 72 h of treatment). In the same study, it was emphasized that even at a higher concentration (1 mg/ml), the extract does not have any cytotoxic activity on normal cell lines (HUVECs) (87).

More recently, several studies were focused on evaluating the cytotoxic activity of an isolated compound from the shallot and some other plant species, namely isoliquiritigenin. The underlying mechanisms involved in anti-tumor effects exerted by this compound as well as the applications of isoliquiritigenin thus far in various therapeutic schemes in the treatment of different cancers, alone or in combination with other drugs are summarized in a comprehensive review paper by Wang et al. (123). The cytotoxic effect of another isolated compound from shallot, namely Cepa2 or alliospiroside A (Figure 3D) was tested on the P3U1 myeloma cancer cell line. It was found that this compound is highly efficient, with a 91.13% reduction in P3U1 cell viability 12 h post-treatment. The reduction of cell viability was correlated with the increase in reactive oxygen species levels in Cepa2-treated P3U1 cells, as compared with untreated cells. Moreover, scanning electron microscope results demonstrated apoptosis of the Cepa2-treated P3U1 cells in a time course-dependent manner (82).

### Anti-angiogenetic Effect

Angiogenesis plays a critical role in cancer development since the proliferation as well as the metastatic spread of cancer cells depends on an adequate supply of oxygen and nutrients (124). Until now, a couple of studies assessed the anti-angiogenetic potential of shallot extracts. The anti-angiogenetic effect of *A. ascalonicum* extract fractions was examined on human umbilical vein endothelial cells (HUVEC) in collagen matrix and chicken chorioallantoic membrane models (47). The study concluded that the ethyl-acetate fraction and one of its subfractions potently inhibited angiogenesis both *in vitro* and *in vivo*, even after treatment in high thermal and low pH conditions (see Figure 2l).

Moreover, another group found that in an aorta ring model that the aqueous extract of *A. ascalonicum* bulbs has noticeable anti-angiogenic activity without toxic effect on the cells in doses that ranged from 50 to 800 μg/ml (104).

### Cancer Prevention

Besides these cytotoxic and anti-angiogenetic effects, shallot extracts are involved in cancer prevention as well. A study published back in 1978 documented that the juice prepared from shallot was found to possess strong capacities of inactivating the mutagenicity of tryptophane pyrolysis products, similar to cabbage, broccoli, green pepper, eggplant, apple, burdock, ginger, pineapple, and mint leaf (103). No other *in vitro* studies were conducted recently on shallot assessing this effect. However, four clinical studies were published recently, investigating the link between shallot and other *Allium* species consumed and the incidence of various types of cancer (i.e., gallbladder cancer, multiple myeloma, gastric cancer, and breast cancer). These studies are briefly discussed below and more details can be found in Table 3.

A study conducted in Shanghai, China on 627 patients, aiming to link the diet with the occurrence of biliary tract cancer demonstrated an inverse association between gallbladder cancer incidence and an *Allium*-based diet (OR: 0.81, 95% CI: 0.68–0.97) (105). In contrast, the study highlights that both preserved vegetables and salted meats food groups showed positive associations with gallbladder cancer. The study failed, however, to link the ampulla or Vater cancer cases with the diet due to the small sample size. On the other hand, another study conducted in China demonstrated that the intake of shallot and garlic was significantly associated with a reduced risk of multiple myeloma (OR = 0.60, 95% CI: 0.43–0.85), similar to soy food and green tea intake (106). The study further reports an increased risk of multiple myeloma in case of intake of brined vegetables and pickles. Sun et al. further documented a correlation between the invasion depth of gastric cancer and CD44v6 expression. It was found that the group with frequent shallot and garlic consumption (≥ 7 times/week vs. none) has a lower expression of CD44v6 (107). However, this study fails to discriminate if the observed effect is exerted by garlic or shallot. It was also demonstrated in a retrospective study conducted on 36,000 females from Maoshan Municipal People's Hospital that shallot consumption has a protective effect against breast cancer incidence (108).

### Remarks and Future Outlooks

In this part, we aimed to summarize and review the main discoveries on cancer prevention and/or treatment exerted by shallot. Although many published studies are dealing with this topic, the data regarding mechanistic insights of isolated compounds from this plant species is scarce. The data collected from several clinical studies point to an inverse correlation between shallot intake and cancer development, so future research should be focused on screening this species for cancer-preventing agents as well as on finding the mechanisms by which these compounds are influencing the metabolic pathways. However, it is remarkable that several studies were focused on elucidating the cytotoxic effects exerted by shallot extracts as well as by the isolated compounds (e.g., Cepa2, isoliquiritigenin), and no cytotoxic effects on normal cells were reported so far. It is also interesting that the great majority of extracts studied are either aqueous or hydroalcoholic, meaning that the polar compounds are responsible for the biological activity reported. Another observation is that among all cytotoxic assays performed, no study employed a positive control so that the extracts' cytotoxic activity cannot be compared to the standard cytotoxic drugs. A comparative study on the effect of solvents with different polarities is needed to better understand whether the cancer prevention effect observed in clinical trials is due to the presence of organosulphur compounds.

## Other Pharmacological Activities

Several studies were conducted to evaluate different other pharmacological activities of shallot extracts (e.g., nephrotoxicity protection, hepatoprotection; anti-tyrosinase, spermatogenesis improvement, etc.). All these findings are summarized in Table 3 and discussed together as follows:

### Hepatotoxicity

**Figure 4:**
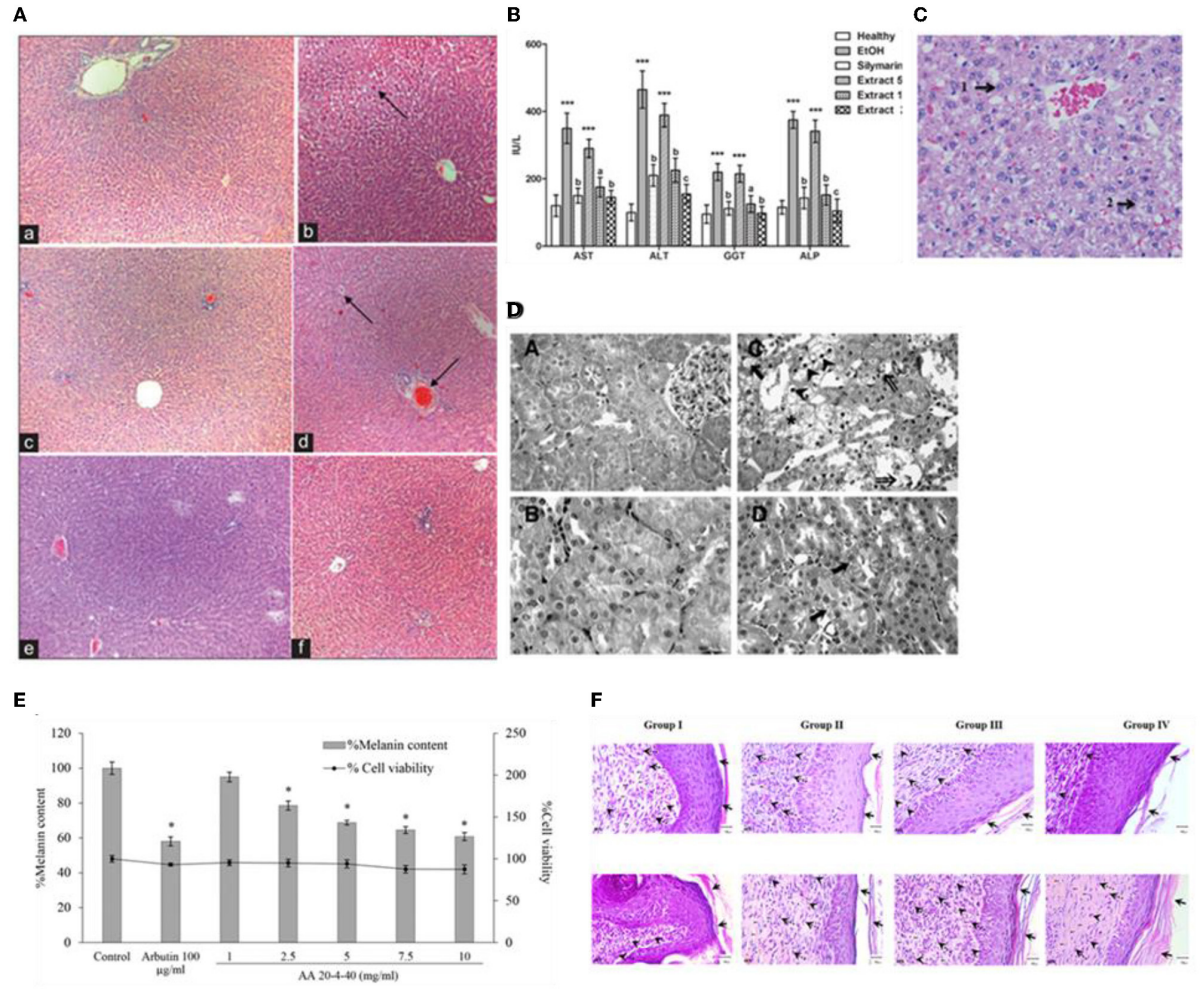
Protective effects of shallot extracts on liver. **(A)** Histological changes in the liver of representative rats of each group (H and E, ×100). (a) Normal control group; (b) hyperuricemic control group; (c) hyperuricemic + 5 mg/kg/day allopurinol; (d) hyperuricemic + 3.5 g/kg/day shallot juice; (e) hyperuricemic + 7.0 g/kg/day shallot juice: (f) hyperuricemic + 10.5 g/kg/day onion juice (110). **(B)** Effect of aqueous crude extract of *A. ascalonicum* bulbs on ethanol-induced liver injury in mice. Groups of mice were administered with 50% (v/v) ethanol for 14 consecutive days followed by treatment with silymarin (10 mg/kg), and extracts (50, 100, and 200 mg/kg), by oral gavage once a day for 7 consecutive days. Liver enzyme activities namely AST, ALT, GGT, and ALP were measured. Normal mice were given only DW and acted as healthy control (125). **(C)** Histopathology of liver cell degeneration in rat K-treated. (1) Normal liver cells. (2) The degenerated liver cells are seen to have swollen so that the cavity looks wider (97). **(D)** Kidney sections from vehicle (A) and shallot extract (B) treated groups, showing essentially normal tubules. CsA treated group (C) showing severe vascular and hydropic degenerative changes. Intracytoplasmic vacuoles (arrow) are present in most of the affected proximal tubular cells. Pyknotic nuclei (arrowhead) and coagulative necrotic cells (asterisk) are evident. Necrotic tubules with dystrophic calcification (double arrow) are observed mainly on the cortico-medullary transitional zone. CsA plus shallot extract treated group (D) showing only multiple small intracytoplasmic vacuoles (111). **(E)** The melanin inhibitory effect of AA 20-4-40 on B16F10 cells. After treatment with AA 20-4-40 (0–10 mg/ml) for 48 h, melanin content and cell viability were measured. The percentage of melanin content was calculated relative to the control group. Arbutin was used as the positive control (109). **(F)** Microscopic view of excision wound healing and epidermal/dermal re-modeling in the pure petroleum gel (Group I), 10% *Allium ascalonicum* extract (Group II), 20% *Allium ascalonicum* extract (Group III), and Terramycin (Group IV) administered animals on days 8 and 14 (20).

An *in vivo* study aiming to investigate the effect of *A. cepa* var. *aggregatum* G. Don juice on serum acid levels of normal and induced hyperuricemic rats concluded that a dose of 10.5 g shallots/kg/day can lower the uric acid levels almost to the same extent as allopurinol (110). Moreover, the research group observed a protective effect of shallot extract on liver damage induced by hyperuricemia (Figure 4A), which was further supported by another study.

Ounjaijean et al. (125) documented a significant, dose-dependent decrease of AST, ALT, GGT, and ALP levels for ethanol-induced liver injury mice treated with *A. ascalonicum* extract (Figure 4B), while Suputri et al. (97) documented that shallot extract can improve histopathological features of rats liver (Figure 4C). However, this last study lacks the details regarding the extraction method employed and shallot origin, thus making it irreproducible.

### Renal Dysfunction

On the other hand, while hepatoprotective effects of shallot are documented, a consensus on the protective effect of shallot against kidney damage is lacking. Wongmekiat et al. showed that the administration of shallot extract along with immunosuppressive cyclosporine A counteracts the deleterious effects of the latter on renal dysfunction, oxidative stress markers, and morphological changes (Figure 4D) (111), while Rahmat et al. (110) observed that shallot extract wasn't able to restore kidney damage produced by hyperuricemia. These apparently contrary results can be explained by the different nephrotoxicity mechanisms exerted by the two studied agents, suggesting that shallot compounds might interact with cyclosporine A damaging mechanism of action, but not with hyperuricemic induced one.

### Skin-Lightening Effects

Recently, many studies were focused on finding alternative skin-lightening agents for the cosmetic industry. Tyrosinase is a key enzyme involved in melanogenesis, thus studies which target it try to discover new compounds with the potential of skin-whitening agents. A study by Phetmanee et al. revealed that the shallots cultivated in Phayao, Thailand exhibited low cytotoxicity and a high tyrosinase inhibitory effect compared with shallot from other cultivation sites in Thailand. The extract also decreased both mushroom tyrosine activity and intracellular tyrosinase activity in B16F10 cells. In addition, the extract significantly decreased melanin content in cells (Figure 4E) (109).

### Wound-Healing Effect

The study by Saenthaweesuk et al. aimed to evaluate the wound-healing activity of the ethanolic extracts of shallots on an excision wound model. The results of the histological evaluation have confirmed remarkable wound-healing activities of shallot extract (Figure 4F). Besides a fully recovered epidermis, active fibroblasts, blood vessels, and collagen fiber were also found in the dermis on day 8, similar to wounds treated with Terramycin ointment (positive control). The negative control showed a fully recovered epidermidis only on day 12, suggesting that shallot extract has the ability to accelerate the wound-healing process (20).

### Mucolytic and Antitussive Activity

The mucolytic activity of shallot extracts by the decrease in the viscosity of the egg albumen was evaluated by Deswati et al. It was found that 25% of shallot extracts exerted a mucolytic activity equivalent to 0.2% N-Acetylcysteine (114). A recent study by Chokpaisarn et al. (115) reported in a pilot pre-post clinical study that PhyllenCit, a herbal anti-cough mixture, containing 3.23% shallot bulbs has a positive effect in suppressing cough and improving the quality of life (QOL) of patients with acute cough. These results confirm the use of shallot in treating cough.

### Nutritional Value

Regarding the nutritional value of shallots, there are conflicting reports on how shallots affect the availability of critically important minerals, specifically iron. Tuntipopipa et al. assessed the effects of common culinary spices and herbs used in the Thai diet (including shallot) on iron availability. It was observed a decrease in iron availability by 50–80% when shallot containing 3–13 mg polyphenols as gallic acid equivalent was added to the meal. This effect was attributed to the shallots' content in phytate (368 ± 12.8 mg/100 g dw), a known binder of minerals that can reduce iron absorption too (117). Contrary to these results, Luo et al. (118) documented that shallot extracts have positively influenced *in vitro* availability of iron and zinc from food grains.

### Anoctamin-1 Inhibitory Activity

Mai et al. isolated ten triterpenoid glycosides from the methanol extract of *A. ascalonicum* rhizomes, which were evaluated for anoctamin-1 (ANO1) inhibitory activity using yellow fluorescent protein reduction assays. The study revealed that among them, two compounds (compounds 2 and 9, Figure 3G) showed moderate ANO1 inhibitory percentages (83).

## Conclusion and Future Outlooks

In this review, we systematically and comprehensively summarized the pharmacological activities exerted by the extracts of shallot (*A. ascalonicum* L.). The main findings regarding the antimicrobial, antioxidant activity, as well as different applications in cardiovascular diseases, diabetes, cancer management and prevention, are briefly described. In addition, the authors critically discussed the main findings, highlighting the future outlooks for each pharmacological application. Given the multitude of research papers published concerning this species as well as the promising pharmacological application of the extracts enriched in biologically active compounds, this is a timely review on this topic.

The research community should conduct more studies to confirm or infirm the observations from the preliminary studies presented herein, by performing more *in vitro* studies followed by *in vivo* ones and, if the results are promising, by clinical trials. Specifically, the stability of compounds found in this species which exert bioactive activities should be further studied.

Finally, we hope that this paper will encourage more research teams to deeply investigate the chemical composition of this species, as well as the molecular mechanisms involved in these empirically observed pharmacological actions.

## Author Contributions

All authors listed have made a substantial, direct, and intellectual contribution to the work and approved it for publication.

## Funding

This work was supported by a grant of the Romanian Ministry of Education and Research, CNCS—UEFISCDI, Project Number PN-III-P1-1.1-PD-2019-1245, within PNCDI III.

## Conflict of Interest

The authors declare that the research was conducted in the absence of any commercial or financial relationships that could be construed as a potential conflict of interest.

## Publisher's Note

All claims expressed in this article are solely those of the authors and do not necessarily represent those of their affiliated organizations, or those of the publisher, the editors and the reviewers. Any product that may be evaluated in this article, or claim that may be made by its manufacturer, is not guaranteed or endorsed by the publisher.
